# Detection of myocardial ischemia by intracoronary ECG using convolutional neural networks

**DOI:** 10.1371/journal.pone.0253200

**Published:** 2021-06-14

**Authors:** Marius Reto Bigler, Christian Seiler

**Affiliations:** Department of Cardiology, Inselspital, Bern University Hospital, University of Bern, Bern, Switzerland; Vellore Institute of Technology: VIT University, INDIA

## Abstract

**Introduction:**

The electrocardiogram (ECG) is a valuable tool for the diagnosis of myocardial ischemia as it presents distinctive ischemic patterns. Deep learning methods such as convolutional neural networks (CNN) are employed to extract data-derived features and to recognize natural patterns. Hence, CNN enable an unbiased view on well-known clinical phenomenon, e.g., myocardial ischemia. This study tested a novel, hypothesis-generating approach using pre-trained CNN to determine the optimal ischemic parameter as obtained from the highly susceptible intracoronary ECG (icECG).

**Method:**

This was a retrospective observational study in 228 patients with chronic coronary syndrome. Each patient had participated in clinical trials with icECG recording and ST-segment shift measurement at the beginning (i.e., non-ischemic) and the end (i.e., ischemic) of a one-minute proximal coronary artery balloon occlusion establishing the reference. Using these data (893 icECGs in total), two pre-trained, open-access CNN (GoogLeNet/ResNet101) were trained to recognize ischemia. The best performing CNN during training were compared with the icECG ST-segment shift for diagnostic accuracy in the detection of artificially induced myocardial ischemia.

**Results:**

Using coronary patency or occlusion as reference for absent or present myocardial ischemia, receiver-operating-characteristics (ROC)-analysis of manually obtained icECG ST-segment shift (mV) showed an area under the ROC-curve (AUC) of 0.903±0.043 (p<0.0001, sensitivity 80%, specificity 92% at a cut-off of 0.279mV). The best performing CNN showed an AUC of 0.924 (sensitivity 93%, specificity 92%). DeLong-Test of the ROC-curves showed no significant difference between the AUCs. The underlying morphology responsible for the network prediction differed between the trained networks but was focused on the ST-segment and the T-wave for myocardial ischemia detection.

**Conclusions:**

When tested in an experimental setting with artificially induced coronary artery occlusion, quantitative icECG ST-segment shift and CNN using pathophysiologic prediction criteria detect myocardial ischemia with similarly high accuracy.

## Introduction

The electrocardiogram (ECG) is an easy available biomedical tool yielding diagnostic information on various cardiac pathologies, specifically acute myocardial ischemia, where the presence or absence of ECG ST-segment shift has therapeutic consequences [1].

However, as myocardial ischemia directly affects *all* energy-dependent cellular processes, several ECG parameters other than ST-segment shift also reflect myocardial ischemia. Recently, our research group employed an experimental setting with complete coronary artery balloon occlusion during 1 minute, thus creating a brief myocardial ischemia [2, 3]. Intracoronary ECG (icECG) was used to assess the diagnostic accuracy of various ECG parameters for ischemia detection [4], whereby the icECG ST-segment shift measured at the J-point at a threshold of 0.365mV was superior to other parameters for ischemia detection. In the mentioned study, selection of the analyzed parameters was based on a literature search for ECG ischemia parameters. Thus, it is likely that potentially well-performing parameters were overlooked based on missing literature.

**Fig 1:**
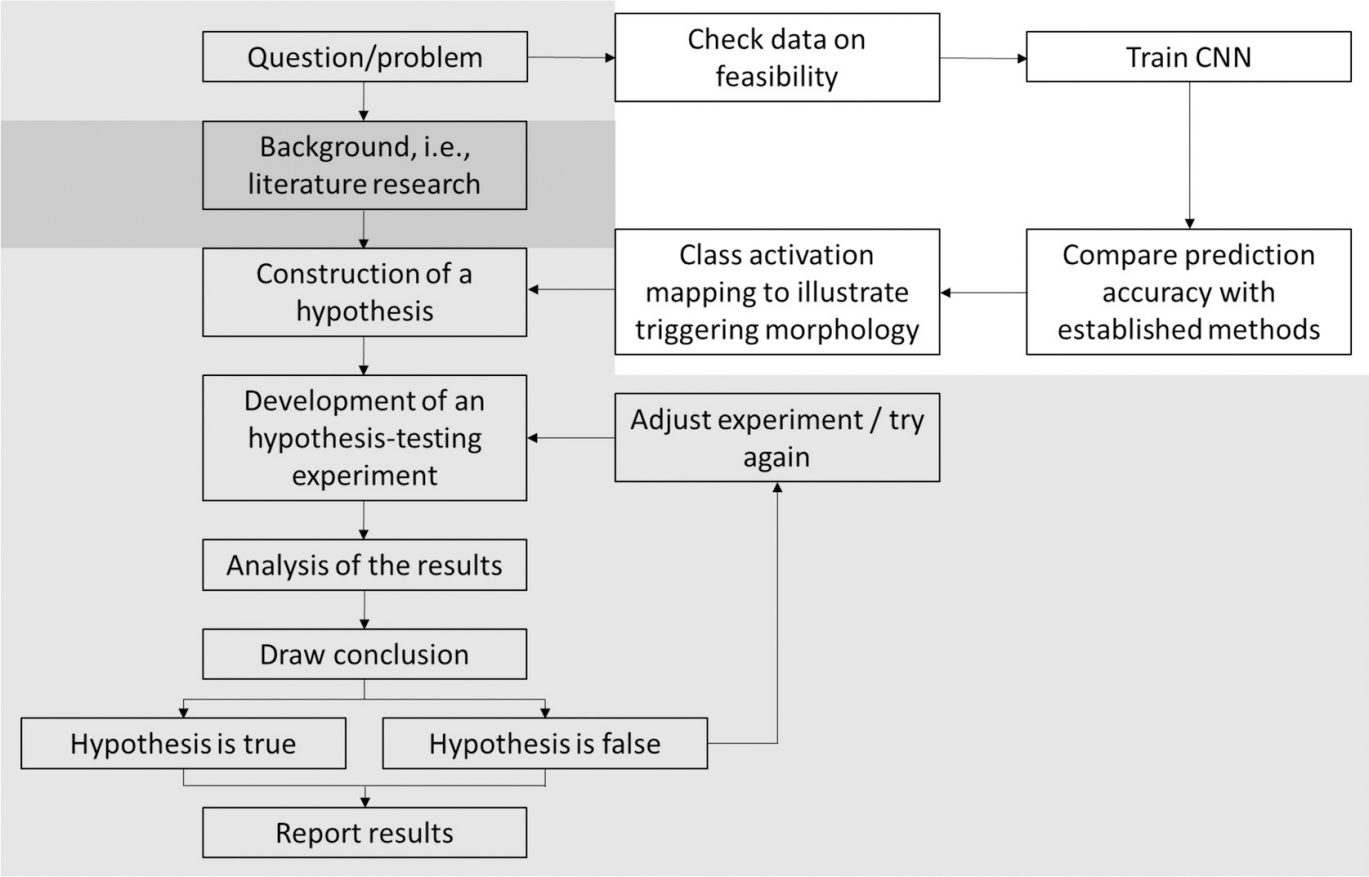
Comparison of the standard scientific method and the deep–learning based approach. Starting from a question/problem to be solved, the standard scientific method is based on a thorough literature research resulting in the construction of a hypothesis. Conversely, the deep–learning based approach uses already available data to train a neural network to solve the defined task as good as possible. Assessment of its performance requires comparison with previously established methods. The best performing networks are then analyzed by class activation mapping to visualize the underlying morphology triggering the network. Based on these visualizations, a hypothesis is constructed and tested in an appropriate experiment. Light–grey: shared processes; dark–grey: standard scientific method; white: deep–learning based, hypothesis–generating approach.

In comparison, deep learning methods, such as convolutional neural networks (CNN) are not affected by a selection bias based on available literature. Instead, the algorithm tries to find patterns in a given dataset to solve a pre-defined task [5, 6]. Hence, CNN focuses on data-derived features rather than on pre-defined parameters, offering an interesting novel approach to enable an unbiased view on well-known clinical phenomenon. Contrary to previous studies [7–13], where CNN processed ECG data to provide a diagnostic aid for clinical physician, this study contributes a novel, deep learning based hypothesis-generating approach applicable from basic science to clinical problems (Fig 1). For the demonstration of the feasibility of this approach, the study implemented transfer learning (i.e., retraining) of pre-trained CNN to determine the optimal icECG parameter for myocardial ischemia detection.

In a first step, multiple CNN were trained to differentiate between non-ischemic and ischemic images of icECG. Then, parametric visualization of the CNN-derived activation patterns was used to find the ECG morphology responsible for the network prediction. Of note, patterns found and used by well-performing CNN to predict the presence or absence of myocardial ischemia were identical to currently used parameters by physicians. Thus, providing an independent validation of these icECG ischemia parameters.

## Methods

### Study design and patients

This was a retrospective observational study in patients with chronic coronary syndrome who underwent coronary angiography due to chest pain, and participated in one of several clinical trials [14–16] our research group carried out between July 2016 and October 2020. As part of all those trials, coronary collateral flow index (CFI) was obtained, i.e., the quantitative measure of coronary collateral function during a brief, artificial coronary artery occlusion. A detailed description of CFI has been previously published [17]. In brief, CFI is a measure of collateral blood supply to a coronary artery proximally balloon-occluded for the duration of 1 minute, and it is defined as mean coronary occlusive pressure relative to mean aortic pressure, both subtracted by central venous pressure [18]. Hence, the present study employs the same temporal landmarks with non-ischemic (i.e., before coronary occlusion), and controlled ischemic (i.e., at the end of the occlusion) conditions as recently [4]. Of note, the experimental setting of a brief artificial coronary balloon occlusion creates an independent reference essential for the subsequent analysis.

Criteria for inclusion in the present analysis were previously conducted CFI measurements with simultaneous recording of icECG, and written informed consent for further use of the patient’s data. Exclusion criteria were the presence of ECG bundle branch blocks, and of non-sinus rhythm or paced rhythm. Application of these criteria resulted in 893 icECG tracings. A patient could thus provide more than two (one non-ischemic and one ischemic) icECG tracings to the data set. IcECGs did not have to be present in pairs of both conditions.

All original studies had been approved by the Ethics Committee of the Canton of Bern, Switzerland, and all patients gave written informed consent for further use of their data. Data collection, storage and analysis was performed retrospective and offline on the local servers of the University Hospital of Bern. Thus, potential issues on data leakage, federated learning or problems concerning real-time transfer of data were not applicable [19–22].

### Acquisition and preparation of the intracoronary ECG

IcECG was acquired by attaching an alligator clamp to the 0.014-inch pressure monitoring angioplasty guidewire (PressureWire™ X Guidewire, Abbott, Chicago, Illinois, United States) positioned in the distal third of a major coronary artery, and connecting it to a precordial lead. The structure of this guidewire with non-conductive coating allows the generation of an icECG-lead between the Wilson Central Terminal and the conductive pressure sensor of the guidewire located near the tip without the need for additional isolation. IcECG recording was performed at a sampling frequency of 2’000 Hz, and with standard system filtering (corresponding to a bandpassfilter 0.05-100Hz). The same guidewire served as angioplasty guidewire for the balloon catheter used for proximal coronary balloon occlusion.

**Fig 2:**
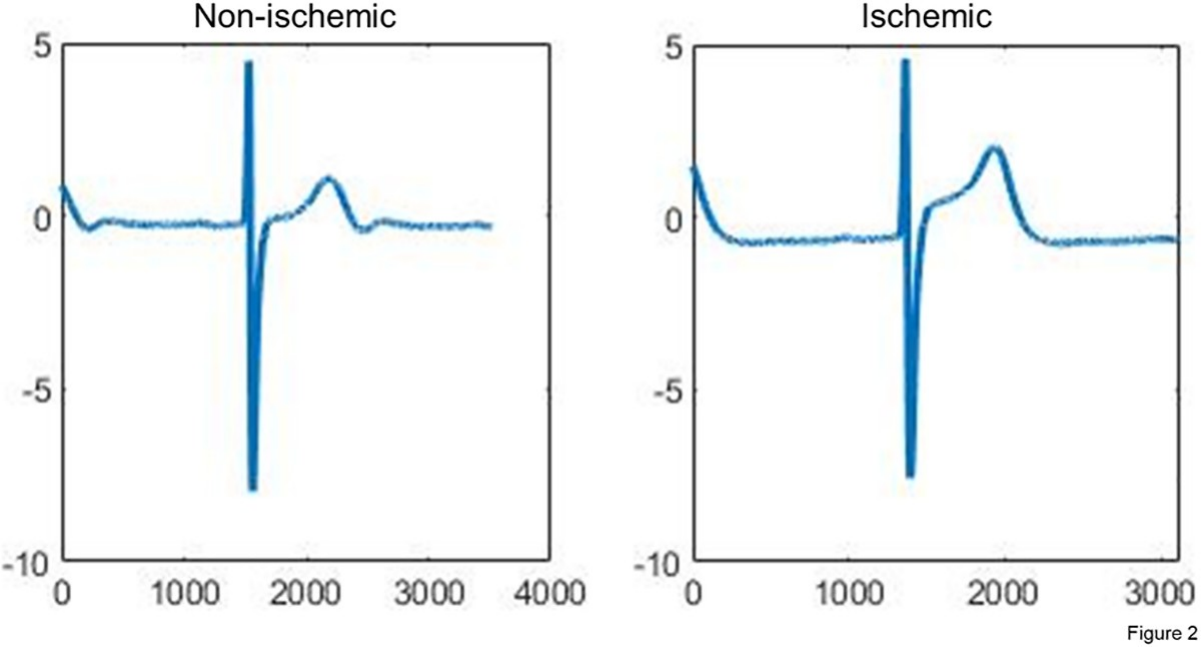
Input data for the neural networks. The input data were taken from the original study analysis and converted into jpgs with a predefined image size (224x224x3 pixels). Each image contained either the illustration of a non–ischemic (i.e., recorded directly before the coronary balloon occlusion) or an ischemic (i.e., recorded at the end of the balloon occlusion) intracoronary ECG as well as the corresponding label (non–ischemic respectively ischemic). In this example, both icECGs are from the same vessel (left anterior descending coronary artery) from the same patient. IcECG ST–segment shift was 0.056mV respectively 0.858mV.

In a subsequent step, 12 to 15 consecutive cardiac cycles were manually chosen according to the intra-procedural tagging as “non-ischemic” or “ischemic” (i.e., recorded during coronary patency respectively coronary occlusion). The chosen cardiac cycles were then signal averaged, additionally plotted in Matlab and saved as jpg-images and stored in group-specific folders (491 non-ischemic, and 402 ischemic icECG images; Fig 2).

### Image allocation and data augmentation

Of the 893 icECG images, 58 were randomly separated into an examination folder for final diagnostic accuracy assessment independent of the training and validation data. The remaining 835 icECG images were randomly allocated to training and validation data (80% respectively 20% as recommended by Goodfellow et al. [5]), the validation data being used to assess the performance of the trained networks during the training process. This resulted in 668 training and 167 validation images. Because of the strong spatial dependence of the icECG with relevant regional morphologic changes within each patient, the likelihood that the network performs overfitting on single patients was judged small. Thus, randomization was not made on a patient level and recordings from two different coronary arteries of the same patient were allowed in two different data sets.

Before the start of each training iteration, all training images were randomly shuffled and processed by adding data noise to prevent overfitting [5, 23]. That is, the images were randomly rotated in a range between ±45°, translocated ±10 pixels in every direction and/or reflected on the horizontal axis.

### Selection and preparation of the pretrained convolutional neural networks

Pretrained CNN were trained on millions of images (for the ImageNet [24] Large Scale Visual Recognition Challenge (ILSVRC; http://www.image-net.org/challenges/LSVRC/), whereby general pattern recognition skills have been already previously developed, thus, allowing the application of a complex network architecture on a small data set. For this study, two CNN with different depth and network architecture were chosen. Both had an input size of 224x224x3 pixels for the images allowing a single data set preparation.

- GoogLeNet (GN) is a 22 convolutional layer deep CNN developed by Szegedy et al. [25] named in honor of the first CNN (LeNet by Yann LeCun [26]). GN has a special architecture with networks within networks (called inception modules). These modules contain multiple different filter sizes allowing simultaneous feature extraction on different levels of details [27].
- ResNet101 (RN) is a 101 convolutional layer deep CNN developed by Kaiming He et al. [28] using a special residual learning framework allowing the training of a deeper and thus more accurate network. As a trade-off, its prediction time is significantly longer as that of GN.

To prepare for the transfer learning process, the last three layers of the networks responsible for the network prediction had to be replaced for the new task, i.e., the classification of icECG images into non-ischemic respectively ischemic. In addition, a dropout layer was added to prevent the network from overfitting [29]. The remaining layers responsible for pattern recognition and feature extraction were not changed. General learning rate was chosen low while the new layers received a learning rate weight factor of 10 (i.e., 10-fold the normal learning rate) to improve and accelerate their training process.

### Transfer learning (CNN training)

During transfer learning, the pretrained CNN were retrained on the new task. For this purpose, the network analysed each image in the training data set and classified it. After n-images (i.e., minibatch size), the network parameters were updated to reflect the new insights learned from the n-images, and the training continued until all images were analyzed once (i.e., one epoch). During this process, several parameters were involved, which had to be determined before training. In this study, GN was used for determining the range of the four hyperparameters, i.e., learning rate, dropout probability, minibatch size and number of epochs. For each hyperparameter, approximately ten training runs were conducted within a broad range of values (e.g., for the dropout probability 0–1 in 0.1 steps) using two optimizer algorithms (stochastic gradient descent with momentum, SGDM [30], and adaptive moment estimation learning rate algorithm, ADAM [31]).

After defining a range of working values for each hyperparameter, 111 training runs using the random search approach [32] were performed using the ADAM optimizer algorithm. A random search was performed only with ADAM because of the better performance in less time. Further, hyperparameter optimization for GN with five cycles of Bayesian optimization [33] (each with 30 training runs, three cycles with SGDM, two cycles with ADAM) was performed resulting in 150 trained networks.

Based on the experience with GN hyperparameter optimization and the concordance of working values for each hyperparameter with the literature, no grid or random search was performed for RN. Instead, optimal setting of the hyperparameter was directly assessed by Bayesian optimization. Because of the significantly longer training duration for RN compared to GN, only two cycles (each with 30 training runs using the ADAM optimizer algorithm) were performed.

### Network performance analysis

Network performance analysis resulted in 321 trained networks (261 GN, 60 RN). Networks performing above the arbitrary threshold of 85% classification accuracy (i.e., (true positive + true negative)/(true positive + true negative + false positive + false negative)) on the validation data were stored for in-depth evaluation with determination of diagnostic accuracy on the validation data, the examination data as well as the combined data sets. Based on the results of this evaluation, the ten best performing networks (independent of the architecture) were further evaluated with class activation mapping (CAM) [34, 35], i.e., parametric visualization of their activation patterns to find the morphology responsible for the network prediction using ten characteristic icECGs (S1–S10 Figs).

### Computational hardware

Network training was simultaneously performed on two computers (Intel® Core™ i7-7700 CPU@3.60GHz, 8GB RAM respectively Intel® Core™ i7-8550U CPU@1.80GHz, 8GB RAM) using customized software (written in Matlab R2019b and R2020a). Average training time during Bayesian optimization was 117 minutes for GN and 186 minutes for RN.

### Statistical analysis

Two study groups based on the temporal landmarks for non-ischemic (i.e., before coronary occlusion) and controlled ischemic (i.e., at the end of the occlusion) conditions were formed. Between-group comparison of continuous study parameters was performed by a paired student’s t-test.

Network performance was analyzed by determination of classification accuracy (i.e., correct classified images/all images) using a 4-field matrix and calculation of sensitivity, specificity and F1-score (harmonic mean of sensitivity and positive predictive value). Nonparametric receiver operating characteristics (ROC) analysis using the reference of coronary patency or occlusion for absent or present myocardial ischemia was performed for accuracy assessment of detecting myocardial ischemia by manually obtained icECG ST-segment shift (continuous) and the CNN prediction (dichotomous). Comparison of the area under the ROC curves was performed using the DeLong-Test.

Statistical significance was defined at a p-level of <0.05. Continuous variables are given as mean ± standard deviation. All analyses were performed using SPSS version 25 (IBM Statistics, Armonk, New York) or MedCalc for Windows, version 19.1 (MedCalc Software, Ostend, Belgium).

## Results

Eight-hundred ninety three icECGs from 228 patients were included in the study. Six-hundred sixty eight were used for CNN training and two-hundred twenty five icECGs for the performance evaluation.

### Patient characteristics

Patient characteristics are presented on Table 1.

**Table 1:** Patient characteristics.

	Overall
Number of patients	228
**Patient characteristics**	
Age (years)	68±10
Female gender (%)	17
Body mass index (kg/m^2^)	28±5
Angina pectoris before intervention (%)	50
Duration of angina pectoris (months)	8±17
Canadian cardiovascular society class of angina pectoris	2.13±0.97
Diabetes mellitus (%)	34
Arterial hypertension (%)	75
Current smoking (%)	17
Cumulative pack years of cigarettes	38±25
Dyslipidemia (%)	80
Family history for coronary artery disease, CAD (%)	29
Prior myocardial infarction (%)	44
**Medical treatment**	
Aspirin (%)	85
Platelet inhibitor (%)	40
Calcium channel-blocker (%)	28
Beta-blocker (%)	61
Nitrate (%)	14
Oral anticoagulation (%)	18
Statin (%)	78
ACE inhibitor or ARB (%)	70
Diuretics (%)	38

### Descriptive statistics

Descriptive statistics of icECG ST-segment shift and the target vessel distribution grouped according to the non-ischemic vs. ischemic state are presented on Table 2. Right coronary artery served most frequent as the study vessel. Manually determined icECG ST-segment shift was different between the groups in each data set.

**Table 2:** ST–segment shift and target vessel distribution.

	Overall	Non-ischemic	Ischemic	p-value
Overall, n	893	491	402	-
ST-segment shift at J-point (mV)	-	0.004±0.300	1.015±0.956	p<0.001
Left anterior descending (LAD), n	278	147	131	p = 0.630
Left circumflex coronary artery (LCX), n	196	107	89	
Right coronary artery (RCA), n	419	237	182	
**Training data**	**668**	**368**	**300**	**-**
ST-segment shift at J-point (mV)	-	-0.007±0.309	1.050±0.950	p<0.001
Left anterior descending (LAD), n	229	119	110	p = 0.456
Left circumflex coronary artery (LCX), n	154	85	69	
Right coronary artery (RCA), n	285	164	121	
**Validation data**	**167**	**92**	**75**	**-**
ST-segment shift at J-point (mV)	-	0.042±0.290	0.931±0.820	p<0.001
Left anterior descending (LAD), n	46	26	20	p = 0.978
Left circumflex coronary artery (LCX), n	35	19	16	
Right coronary artery (RCA), n	86	47	39	
**Examination data**	**58**	**31**	**27**	**-**
ST-segment shift at J-point (mV)	-	0.026±0.197	0.870±1.326	p = 0.003
Left anterior descending (LAD), n	3	2	1	p = 0.876
Left circumflex coronary artery (LCX), n	7	3	4	
Right coronary artery (RCA), n	48	26	22	
**Validation + Examination data**	**225**	**123**	**102**	**-**
ST-segment shift at J-point (mV)	-	-0.038.±0.269	0.915±0.972	p<0.001
Left anterior descending (LAD), n	49	28	21	p = 0.897
Left circumflex coronary artery (LCX), n	42	22	20	
Right coronary artery (RCA), n	134	73	61	

**Fig 3:**
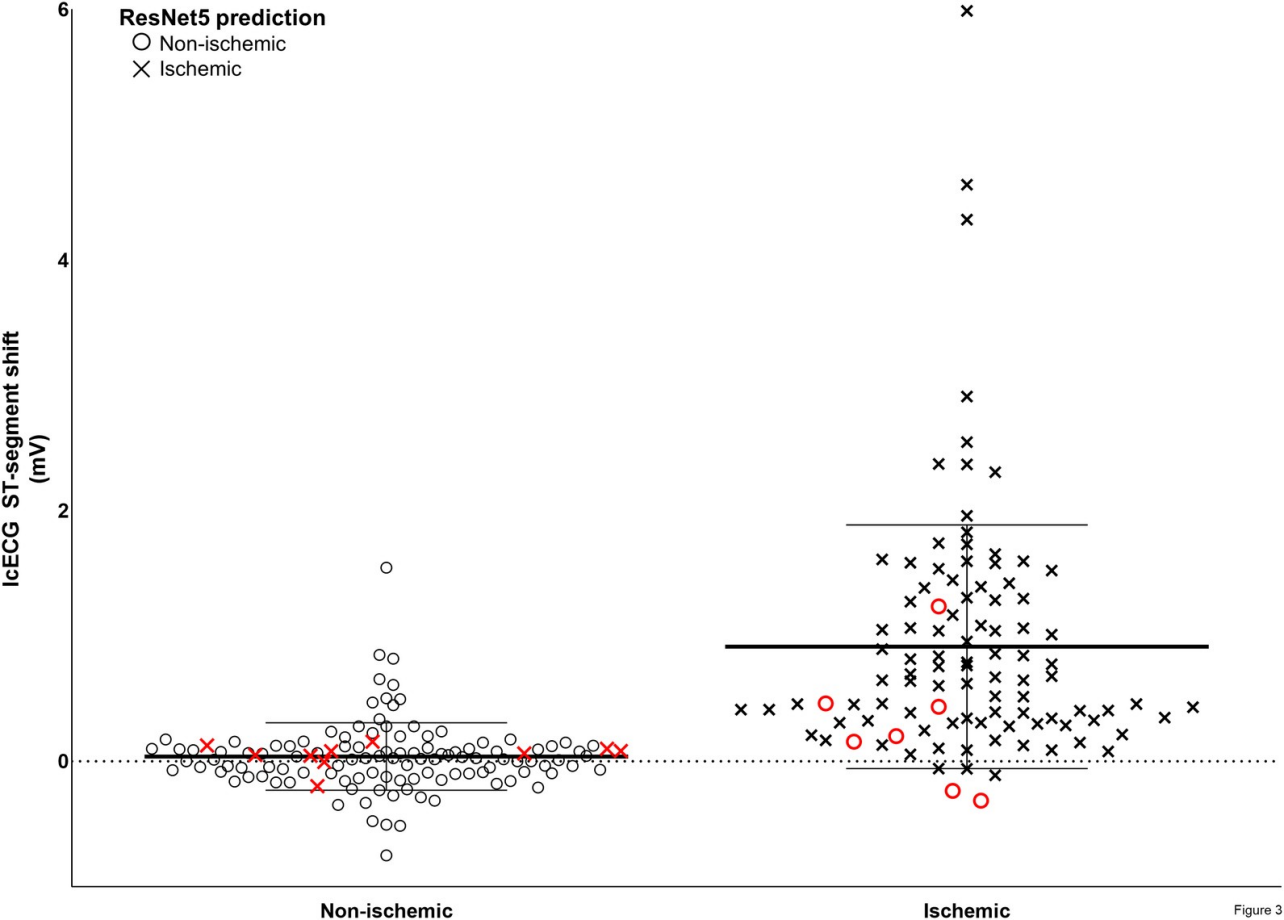
IcECG ST–segment shift grouped according to the state of absent or present coronary artery balloon occlusion. Combination of the validation and the examination data (n = 225) was used for the performance analysis. Black circles: Non–ischemic prediction of ResNet5, black crosses: Ischemic prediction of ResNet5. Red signals: wrong predictions of ResNet5. Error bars indicate mean values and SD.

Fig 3 shows the distribution of icECG ST-segment shift grouped according to the state of absent or present coronary artery balloon occlusion for the combination of validation and examination data as well as the corresponding network prediction from a selected trained network (RN5).

### Receiver-operating characteristic curves

**Fig 4:**
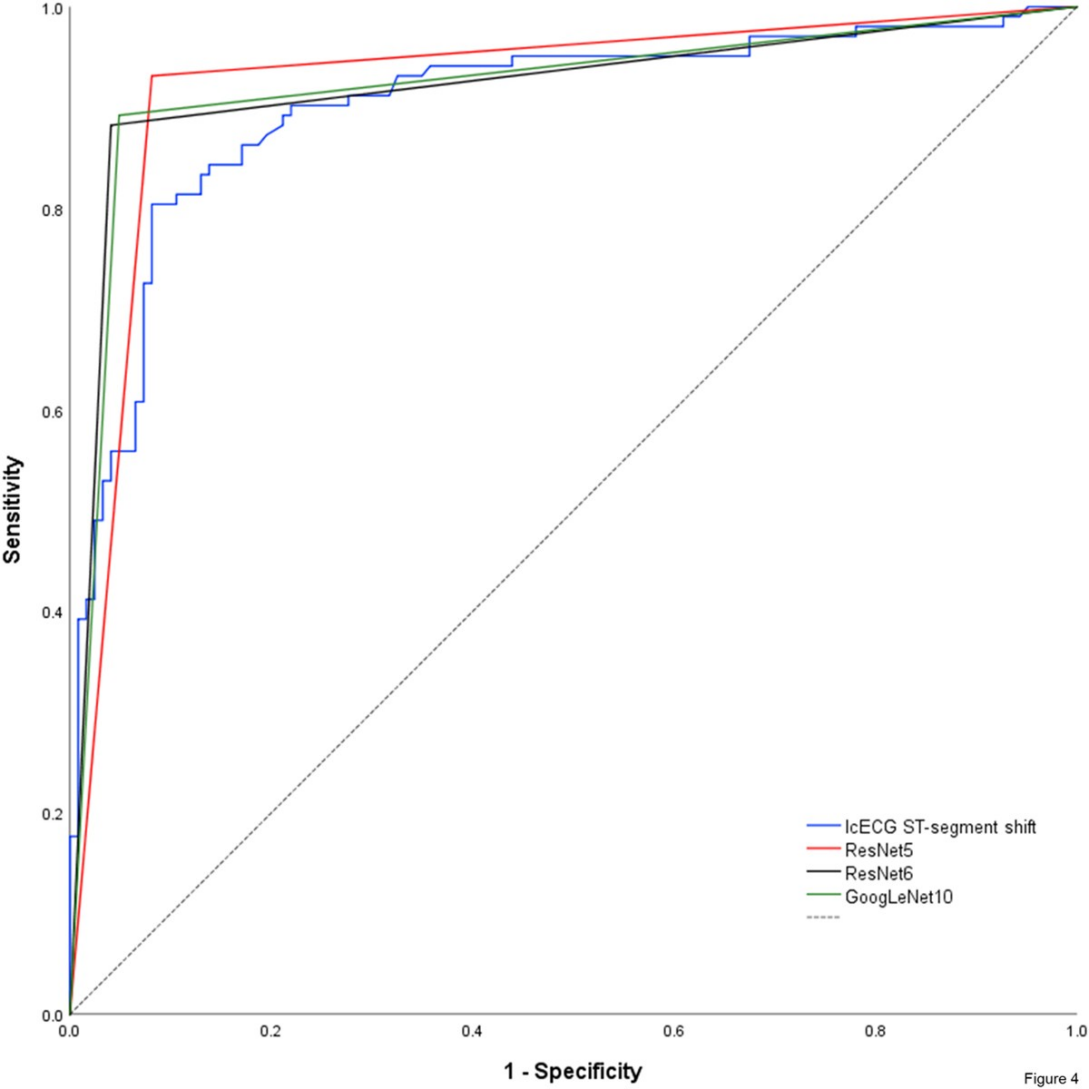
Nonparametric receiver–operating characteristic curve of the icECG ST–segment shift and the network predictions using coronary artery patency or occlusion as dichotomic reference for absent or present myocardial ischemia. Of note, network prediction provides a dichotomous output (non–ischemic respectively ischemic), resulting in a triangular ROC–curve. Hence, there is only one combination of sensitivity and specificity possible for each CNN. Dashed black line = reference line.

Using coronary artery patency or occlusion as dichotomic reference for absent or present myocardial ischemia, receiver-operating-characteristics (ROC) analysis of manually obtained icECG ST-segment shift in mV showed an area under the ROC-curve for the combined validation and examination data of 0.903±0.043 (p<0.0001; Fig 4). AUC for the training data was 0.941±0.019 (p<0.0001), for validation data 0.897±0.051 (p<0.0001), for examination data 0.927±0.074 (p<0.0001) and for the complete data 0.932±0.018 (p<0.0001, S1 Table).

Regarding the optimum cut-off for ischemia detection, an icECG ST-segment shift of 0.279mV distinguished best between non-ischemic and ischemic myocardium, sensitivity 80%, specificity 92% for the combined validation and examination data.

### Network performance

**Fig 5:**
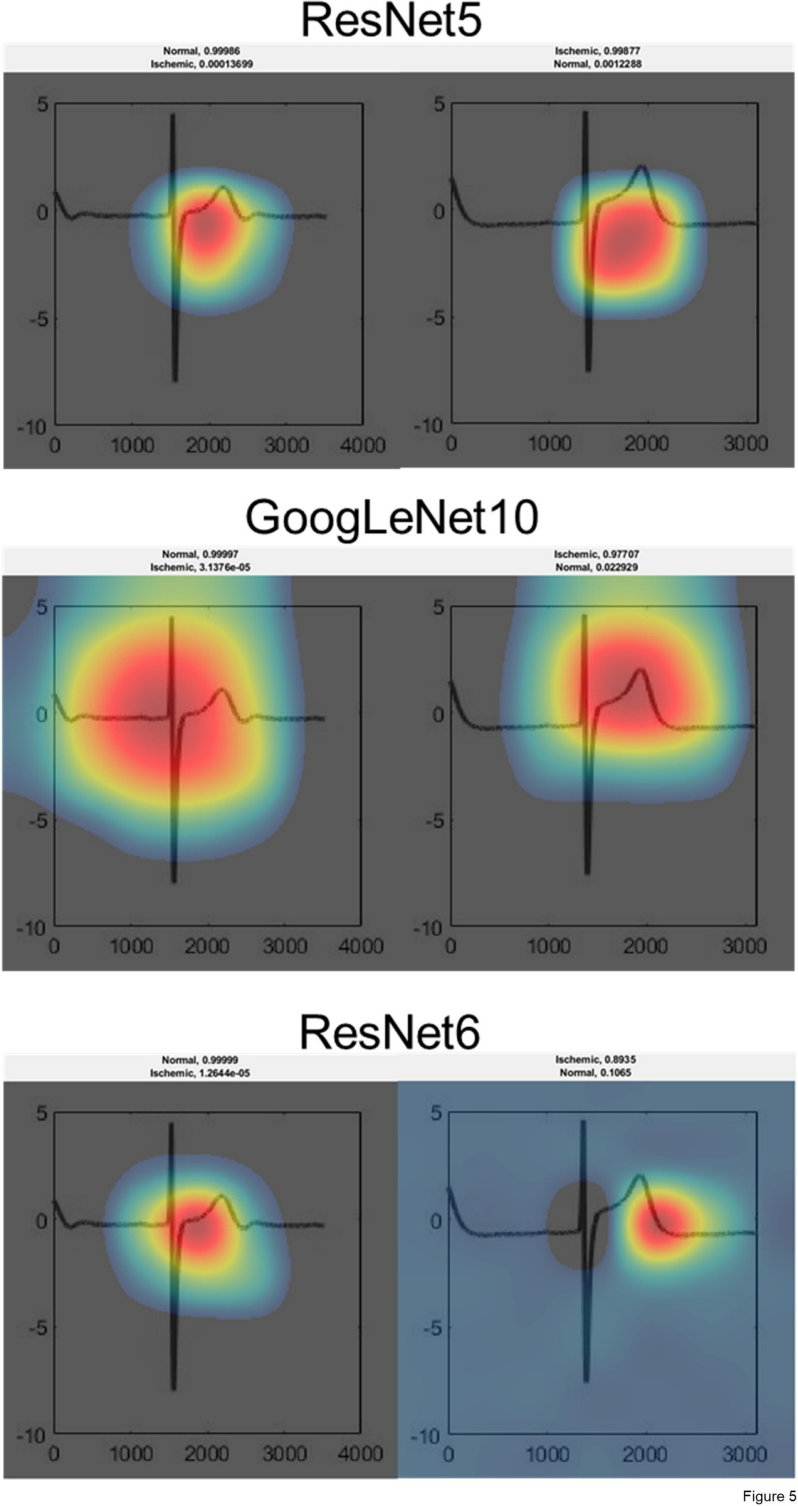
Visualization of network activation patterns of the three best performing CNN. Red regions contributed most to the network class prediction. ResNet5 bases its prediction on the area under the ST–segment and the T–wave. GoogLeNet10 is activated by the QRS–complex and the J–point for the non–ischemic state, and by the ST–segment and the T–wave for the ischemic state. ResNet6 bases its prediction on the ST–segment for the non–ischemic state, and on the end of the T–wave for the ischemic state. Please note the rather uncertain prediction of ResNet6 on the ischemic ECG.

Prediction of the ten best performing networks and their accuracy, sensitivity, specificity and network-activating icECG morphology are presented on Table 3, and in detail on S2 Table. Using the reference (i.e., temporal landmarks before respectively at the end of a coronary balloon occlusion) for absent or present myocardial ischemia, the three best-performing networks showed a diagnostic accuracy of 92% (RN5: sensitivity 93%, specificity 92%; RN6: sensitivity 88%, specificity 96%; GN10: sensitivity 89%, specificity 95%; Fig 4). Visualization of the network activation patterns are shown on Fig 5, and in detail on S1–S10 Figs.

**Table 3:** Prediction and performance of the best ten trained networks.

Convolutional neural network	*True Predicted*	Non-ischemic	Ischemic	Accuracy	Sensitivity	Specificity	F1-score	Activating Morphology
ResNet5: L1e-4_D0.80_M14_E60	Non-ischemic	113	7	92.44	93	92	0.918	Area under the ST-segment and the T-wave
	Ischemic	10	95					
GoogLeNet10: L1e-4_D0.26_M18_E28	Non-ischemic	117	11	92.44	89	95	0.915	QRS + J-point
	Ischemic	6	91					ST-segment + T-wave
ResNet6: L2e-4_D0.52_M25_E60	Non-ischemic	118	12	92.44	88	96	0.914	ST-segment
	Ischemic	5	90					End of T-wave
GoogLeNet2: L1e-4_D0.25_M18_E20	Non-ischemic	114	9	92.00	91	93	0.912	QRS + J-point
	Ischemic	9	93					Mainly J-Point
ResNet7: L1e-4_D0.60_M14_E60	Non-ischemic	116	11	92.00	89	94	0.910	Mainly T-wave
	Ischemic	7	91					
GoogLeNet8: L1e-4_D0.45_M18_E25	Non-ischemic	119	14	92.00	86	97	0.907	QRS + J-point
	Ischemic	4	88					Mainly J-Point
GoogLeNet12: L1e-4_D0.6_M15_E20	Non-ischemic	120	15	92.00	85	98	0.906	Small QRS
	Ischemic	3	87					J-Point
ResNet10: L1e-4_D0.63_M12_E60	Non-ischemic	113	9	91.56	91	92	0.907	ST-segment
	Ischemic	10	93					T-wave
ResNet8: L1e-4_D0.64_M22_E60	Non-ischemic	121	17	91.56	83	98	0.899	J-point/ST-segment
	Ischemic	2	85					T-wave
GoogLeNet13: L1e-4_D0.3_M20_E30	Non-ischemic	121	17	91.56	83	98	0.899	QRS
	Ischemic	2	85					Mainly T-wave

Order according to accuracy. L = learning rate, D = dropout rate, M = minibatch size, E = number of epochs.

Please note that all ResNet–networks were trained with a preliminary termination term (thus, all had E60 but were automatically stopped by the training algorithm).

### Comparison of icECG ST-segment shift and network performance

DeLong-Test of the ROC-curves (Fig 4) showed no significant difference of AUCs between manually obtained icECG ST-segment shift and the three networks (RN5: p = 0.384; RN6: p = 0.435; GN10: p = 0.438). There was no significant difference either between the AUCs among the three networks.

## Discussion

When tested in an experimental setting with systematically induced, complete coronary balloon occlusion, thus establishing the reference of non-ischemic and ischemic myocardium, these conditions are distinguishable by icECG with equal accuracy using manually determined icECG ST-segment shift and convolutional network analysis. The underlying morphology responsible for the network prediction differs between the trained networks, but for myocardial ischemia detection focuses mainly on the icECG ST-segment and the T-wave.

### Assessment of myocardial ischemia by intracoronary ECG

Slightly different from previously published results, icECG ST-segment shift showed a lower diagnostic accuracy (0.903±0.043 for the evaluation data versus 0.963±0.029 [4]), and was less pronounced during myocardial ischemia in the current analysis (0.915±0.972mV vs. 1.272±0.998mV). This resulted in a lower optimal cut-off level for ischemia detection at 0.279mV (compared to 0.365mV). This difference is related to biological and not statistical variance between the recent and the actual investigation. The extent of myocardial ischemia, i.e., the cause of icECG ST-segment shift, depends on multiple factors, namely duration of coronary occlusion, ischemic area at risk for infarction, myocardial oxygen consumption and coronary collateral blood supply [36]:

$$Extent\;of\;icECG\;ST‐segment\;shift≙\frac{Duration\;of\;occlusion*Area\;at\;risk*Myocardial\;oxygen\;consumption}{Coronary\;collateral\;blood\;supply}$$

≙:beingrelatedto

While the duration of coronary artery occlusion was identical in the recent and the present study (i.e., 60 seconds), the other determinants of myocardial ischemia differed. Compared to the previous analysis [4], where left anterior descending coronary artery was the most frequent study vessel, the right coronary artery currently served as the most frequent study vessel, causing a relevant decrease in the myocardial area at risk.

In addition, considering the higher heart rate during myocardial ischemia in the previous study (95±25 vs 72±14 beats per minute), it can be assumed that myocardial oxygen consumption (resulting from ventricular wall stress, heart rate and contractility) was increased as well. Last, coronary collateral function presently did not serve as an exclusion criterion. Accordingly, CFI was significantly higher in the present than the former analysis: 0.123±0.081 vs. 0.084±0.055 (p<0.001).

### Application of CNN on icECG

Based on the ubiquitous availability of the ECG in clinical practice, and together with the rise of wearable devices, an increasing amount of ECG data is available. Effective processing of these data by physicians is not feasible even if it could provide valuable information. Thus, several studies used CNN to process ECGs, primarily with the intention of classifying them according to their rhythm [7–10, 37, 38]. Further, one study has demonstrated that CNN trained on a per-patient level may be an interesting, low-computational possibility to automatically monitor long-term continuous ECG data (e.g., output of implantable devices) [11].

Aside from arrhythmia detection, CNN has also been used to distinguish between other cardiac pathologies. van de Leur et al. used a huge data set with >300’000 ECG recordings to train a CNN to perform a triage into four categories (normal, not acutely abnormal, subacutely abnormal and acutely abnormal) [12]. A panel of five cardiologist served as the reference. Deep neural network in that study demonstrated an excellent overall discrimination with an AUC of 0.93. So called class activation mapping has shown that the network based its predictions on the same regions in the ECG as would physicians [12].

Makimoto et al. trained a small 6-layered CNN on 289 images of a 12-lead ECG resulting in a comparable capability as physicians in recognizing myocardial ischemia on ECG [13]. Visualization of the activation patterns revealed that the CNN was triggered by elevated ST-T-segments. However and as stated by the authors, conclusions of this visualization have to be drawn carefully, as it has been shown that class activation mapping can fail to properly localize objects in an image if the image contains multiple occurrences of the same classification (as it is the case in a 12-lead ECG) [13]. Last, Cohen-Shelly et al. showed the feasibility of artificial intelligence based screening for aortic valve stenosis using standard 12-lead ECG [39].

### Comparison of CNN and manual icECG ST-segment shift measurement

This study demonstrated that transfer learning of a pretrained CNN is highly accurate for detecting myocardial ischemia as reflected by icECG. CNN focuses on distinctive features in the icECG ST-segment and the T-wave. The pathophysiologic basis of these characteristic patterns is the reduced resting potential of the ischemic myocardial cells, caused by a pathologic ion current across the injured cell membrane with subsequent distortion of the normally isoelectric ST-segment [4, 40]. In addition, inadequate energy supply during ischemia directly affects the ventricular repolarization and thus, the morphology and duration of the T-wave [40]. As shown by our previous study, icECG ST-segment shift measured at the J-point outperformed all other icECG parameters in differentiating between non-ischemic and ischemic icECG tracings (area under the ROC curve of 0.963±0.029 vs 0.811±0.057 for amplitude of the T-wave [4]). Hence, it is remarkable that CNN, which focused on the same ECG morphologies revealed a trend to even higher diagnostic accuracy than manually obtained icECG ST-segment shift for ischemia detection.

As compared to icECG ST-segment shift, CNN were not limited to a single, “hand-picked” but to all icECG parameters. Hence, combination of two characteristics, e.g., ST-segment and T-wave integral, enabled the numerically higher diagnostic accuracy. Most CNN used one morphology for the non-ischemic (e.g., QRS-complex), and another one for the ischemic images. Thus, they were also able to differentiate between the physiologic and pathophysiologic state of ischemia on a larger scale.

IcECGs recorded in the RCA are especially challenging as they often show a large atrial signal (P-wave), unstable isoelectric lines and negative T-waves (S1–S10 Figs, middle image in both rows). Further, recording of the icECG in the proximal and mid RCA assesses myocardial ischemia in the low-mass right ventricle (i.e., small ischemic signal) further complicating ischemia detection. Thus, it is not astonishing that 14 out of the 17 (82%) falsely classified images of RN5 were recorded in the RCA, the latter of which represented 60% of the data (see S11 Fig for all falsely classified icECGs of RN5). Nevertheless, and despite the high proportion of RCA icECG recordings, CNN were able to distinguish between non-ischemic and ischemic images with high accuracy demonstrating the robustness of the method.

### Limitations

The biggest limitation of the application of CNN on various problems lies in the very nature of neural networks, i.e., “its complicated interconnected hierarchical representations of the training data to produce its predictions” [41] on unseen data. Thus, interpretation of these predictions is challenging and often referred to as a “black box” problem [41]. While class activation mapping offers a visualization of the activation patterns and enables conclusions on the general distinction procedure, prediction can remain unexplainable. S11 Fig demonstrates all falsely predicted icECG images of RN5. Most of them are understandable, e.g., ischemic morphology of a non-ischemic icECG recorded in the RCA. However, some predictions are incomprehensible. Track-down of the activation pattern in each convolutional layer would possibly enlighten the erroneous predictions. However, such a process within a network architecture with numerous convolutional layers is extremely complex and time-consuming.

Further, the used networks were exceeding the complexity of the presented task by far. This was, however, on purpose, as the objective of our study was to demonstrate the feasibility of a hypothesis-generating process. Thus, to ensure generalizability to other, future tasks, highly capable pretrained networks were used in combination with excessive data augmentation to prevent overfitting. Hence, this method does not provide the most appropriate and efficient CNN, but rather a well-performing CNN to allow generating hypotheses, which need further verification. In addition, the presented task (i.e., to distinguish between non-ischemic and ischemic icECG tracings), would have been possible with raw icECG signal data without the conversion into jpg-images associated with an increase of data points and a loss of details. In this case, pretraining would have been feasible with open source ECG databases. However, this approach would limit the generalizability of the proposed, deep learning approach, as the requirements are not ubiquitous applicable.

While transfer learning of pretrained CNN enabled the application of networks with a high capacity on a small data set, this approach determines also the format of the input data. Thus, data has to be images with restricted input size, which limits the resolution. A requirement, which could cause the missing of subtle patterns. In addition, quantitative assessment is not possible using this deep learning approach and it requires a certain accordance between the original task and the problem to be addressed. As this requirement is not always given, it is possible that the presented approach does not provide accurate network predictions. However, taken into account the relatively low effort and the potentially valuable information, we recommend to try this approach at least once on a particular problem.

Finally, ROC-analysis with binary predictors (i.e., the dichotomous classification by the CNN) is a potential misleading metric [42]. However, the ROC-analysis was not the only statistical assessment of the network performance and all (consistent) results were shown.

### Implications

This study demonstrates the feasibility of a hypothesis generating process using transfer learning of pretrained CNN on a small (n<1000) data set. Contrary to previously conducted ECG studies using deep learning, the goal of this study was not to develop a diagnostic aid for physicians in daily clinical practice, but rather to enable an unbiased view on a well-known clinical phenomenon, i.e., myocardial ischemia. Implementation of this view did not require big data or specialized computational hardware. Instead, a small but well-defined data set was used to perform transfer learning on pretrained CNN using single CPU computers. Hence, the proposed approach is feasible without extensive computational hardware and for a wide variety of scientific problems. Conditions are the presence of a well-defined, independent reference (absent vs present ischemia), and the possibility to transform the data into images (as most of the high capacity, open-available CNN were trained on the ImageNet [24] database).

In the present study, data-derived features used by the CNN to distinguish between absent or present myocardial ischemia were similar to the common practices and focused mainly on the icECG ST-segment and the T-wave. Hence, the hypothesis-generating process did not provide unknown ischemic patterns but rather confirmed the common parameters used to quantify myocardial ischemia in the icECG.

## Conclusion

When tested in an experimental setting with artificially induced coronary artery occlusion, quantitative icECG ST-segment shift and CNN using pathophysiologic prediction criteria detect myocardial ischemia with similarly high accuracy. Thus, this study contributes a novel, deep learning based hypothesis-generating approach applicable from basic science to clinical problems.

## Supporting information

S1 Fig Visualization of network activation patterns of ResNet5. Red regions contributed most to the network class prediction.
(TIF)

S2 Fig Visualization of network activation patterns of GoogLeNet10. Red regions contributed most to the network class prediction.
(TIF)

S3 Fig Visualization of network activation patterns of ResNet6. Red regions contributed most to the network class prediction.
(TIF)

S4 Fig Visualization of network activation patterns of GoogLeNet2. Red regions contributed most to the network class prediction.
(TIF)

S5 Fig Visualization of network activation patterns of ResNet7. Red regions contributed most to the network class prediction.
(TIF)

S6 Fig Visualization of network activation patterns of GoogLeNet8. Red regions contributed most to the network class prediction.
(TIF)

S7 Fig Visualization of network activation patterns of GoogLeNet12. Red regions contributed most to the network class prediction.
(TIF)

S8 Fig Visualization of network activation patterns of ResNet10. Red regions contributed most to the network class prediction.
(TIF)

S9 Fig Visualization of network activation patterns of ResNet8. Red regions contributed most to the network class prediction.
(TIF)

S10 Fig Visualization of network activation patterns of GoogLeNet13. Red regions contributed most to the network class prediction.
(TIF)

S11 Fig Visualization of network activation patterns of all false prediction of ResNet5. Red regions contributed most to the network class prediction. While some predictions are understandable (left and right column), others are incomprehensible (middle column).
(TIF)

S1 Table Nonparametric receiver-operating characteristic curves of manually determined icECG ST-segment shift and corresponding collateral flow index and heart rate. (DOCX)

S2 Table In-detail performance of the ten best performing networks. (DOCX)

S1 Data Raw data, i.e., icECG tracings, used for the conduction of the study. (ZIP)

S1 Graphical abstract (TIF)
