# Supplementary figures and images for: Detection of myocardial ischemia by intracoronary ECG using convolutional neural networks (part 1 of 3)
Source: PLoS One. 2021 Jun 14;16(6):e0253200. doi: 10.1371/journal.pone.0253200 (PMC8202932; doi:10.1371/journal.pone.0253200)

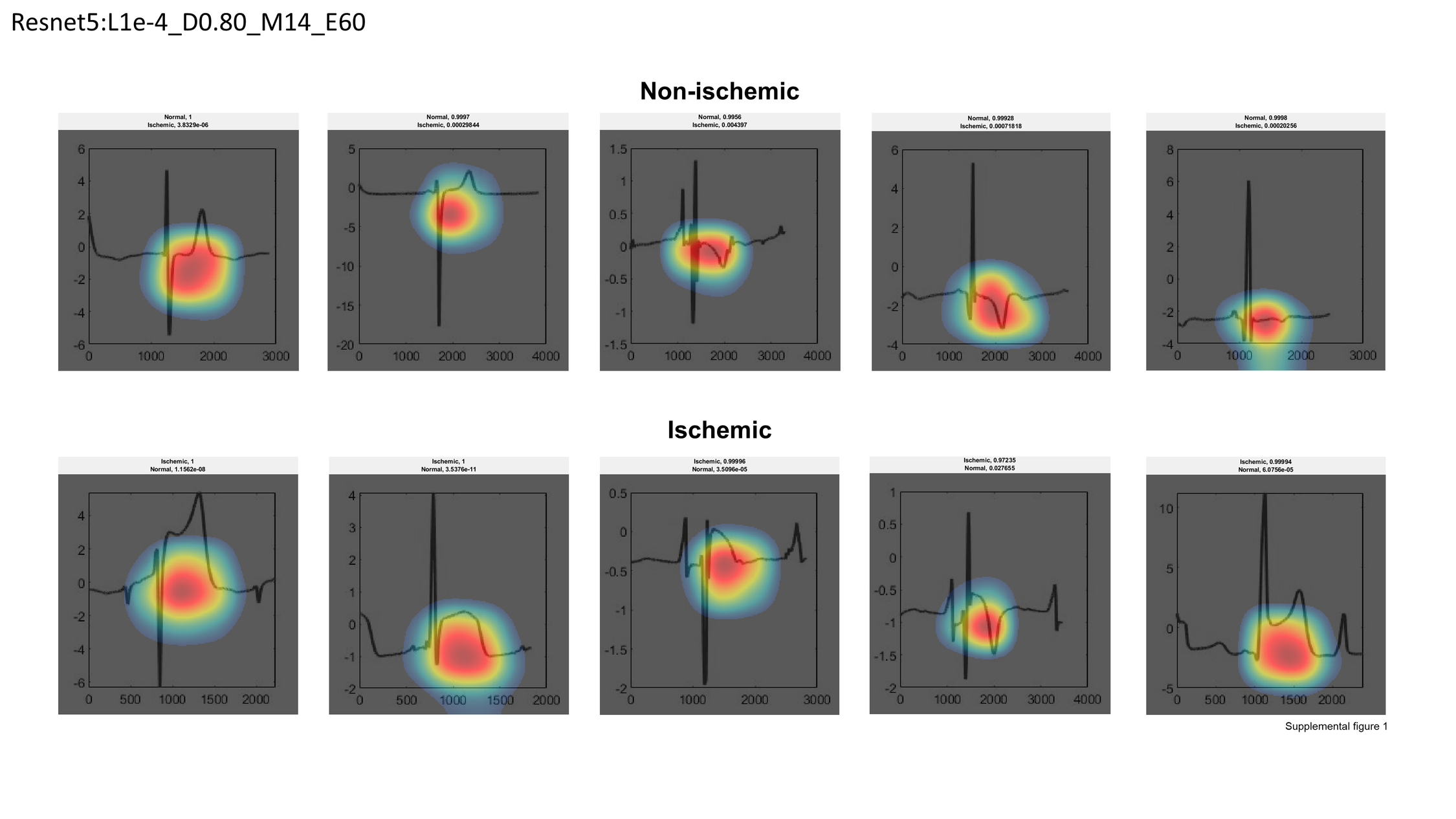

Supplement: S1 Fig — Red regions contributed most to the network class prediction. (TIF) [file pone.0253200.s001.tif]

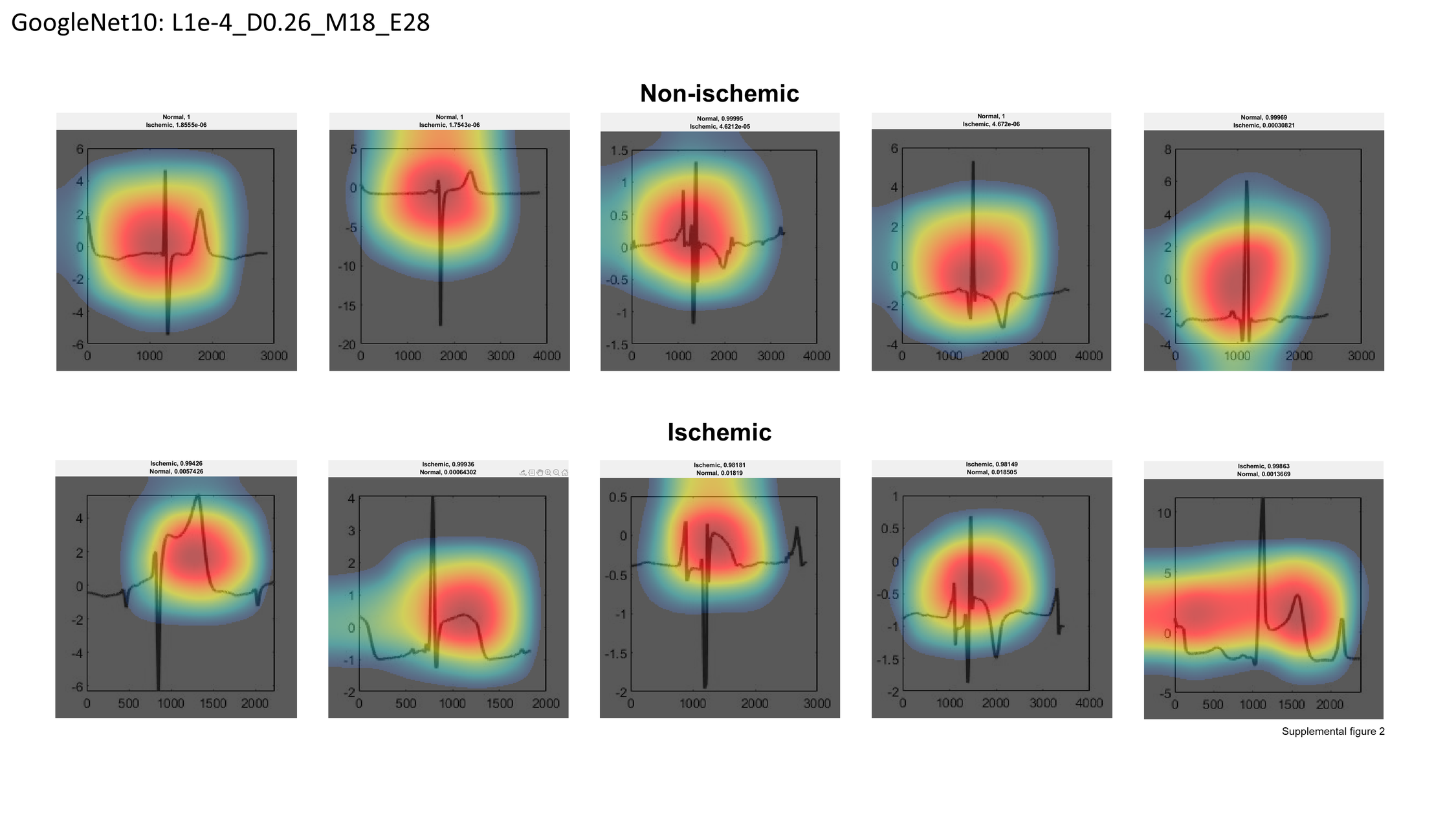

Supplement: S2 Fig — Red regions contributed most to the network class prediction. (TIF) [file pone.0253200.s002.tif]

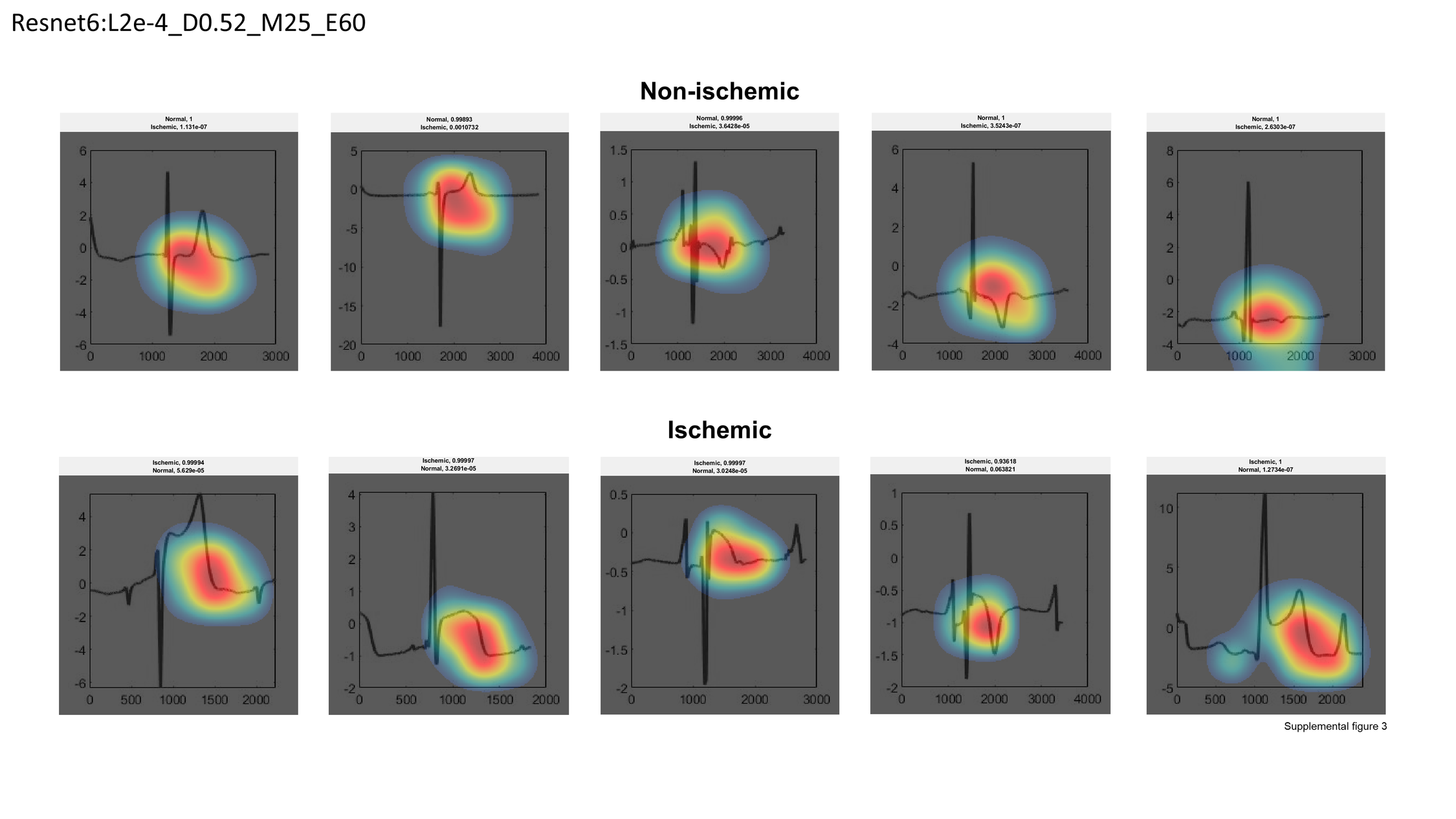

Supplement: S3 Fig — Red regions contributed most to the network class prediction. (TIF) [file pone.0253200.s003.tif]

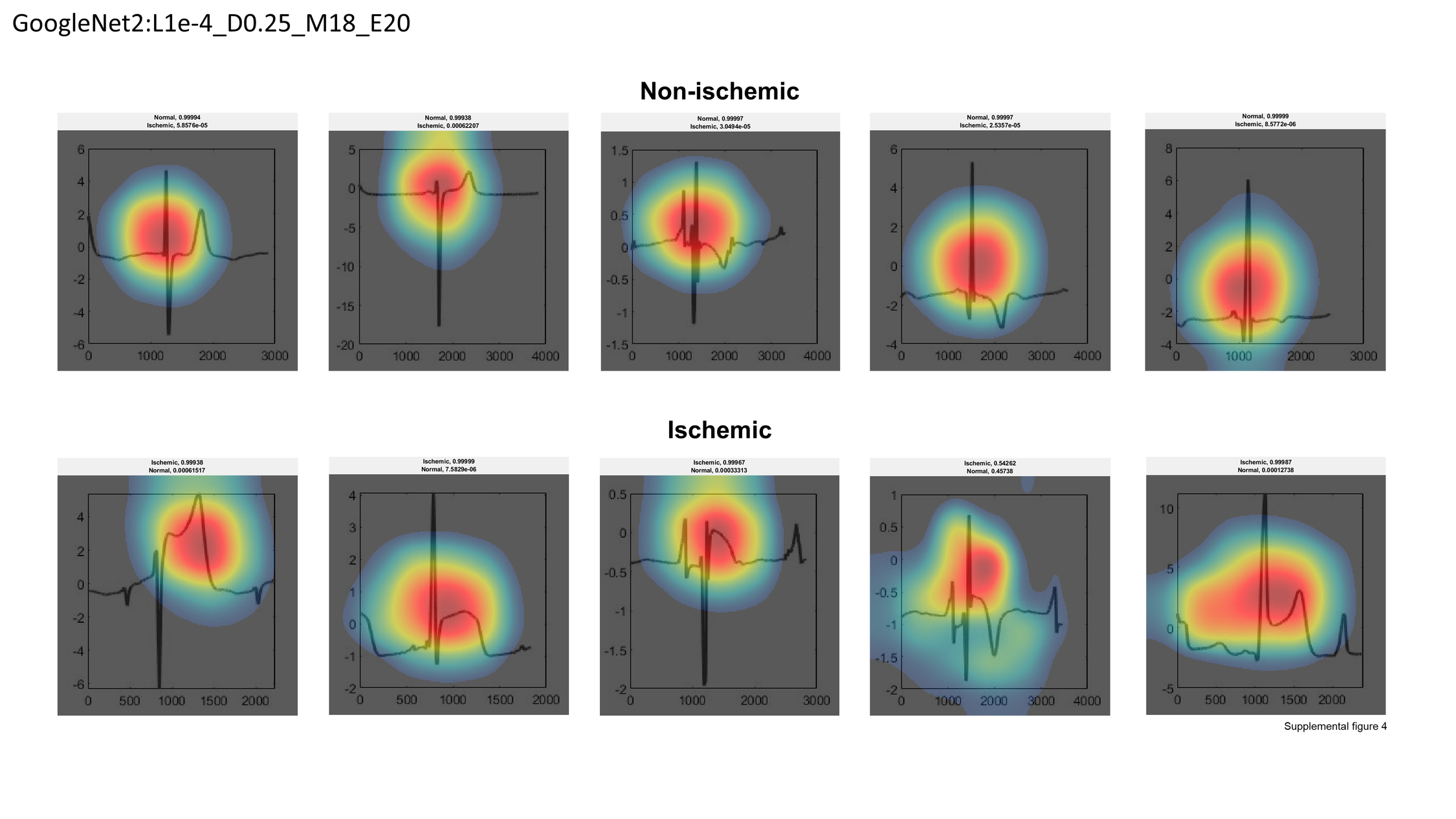

Supplement: S4 Fig — Red regions contributed most to the network class prediction. (TIF) [file pone.0253200.s004.tif]

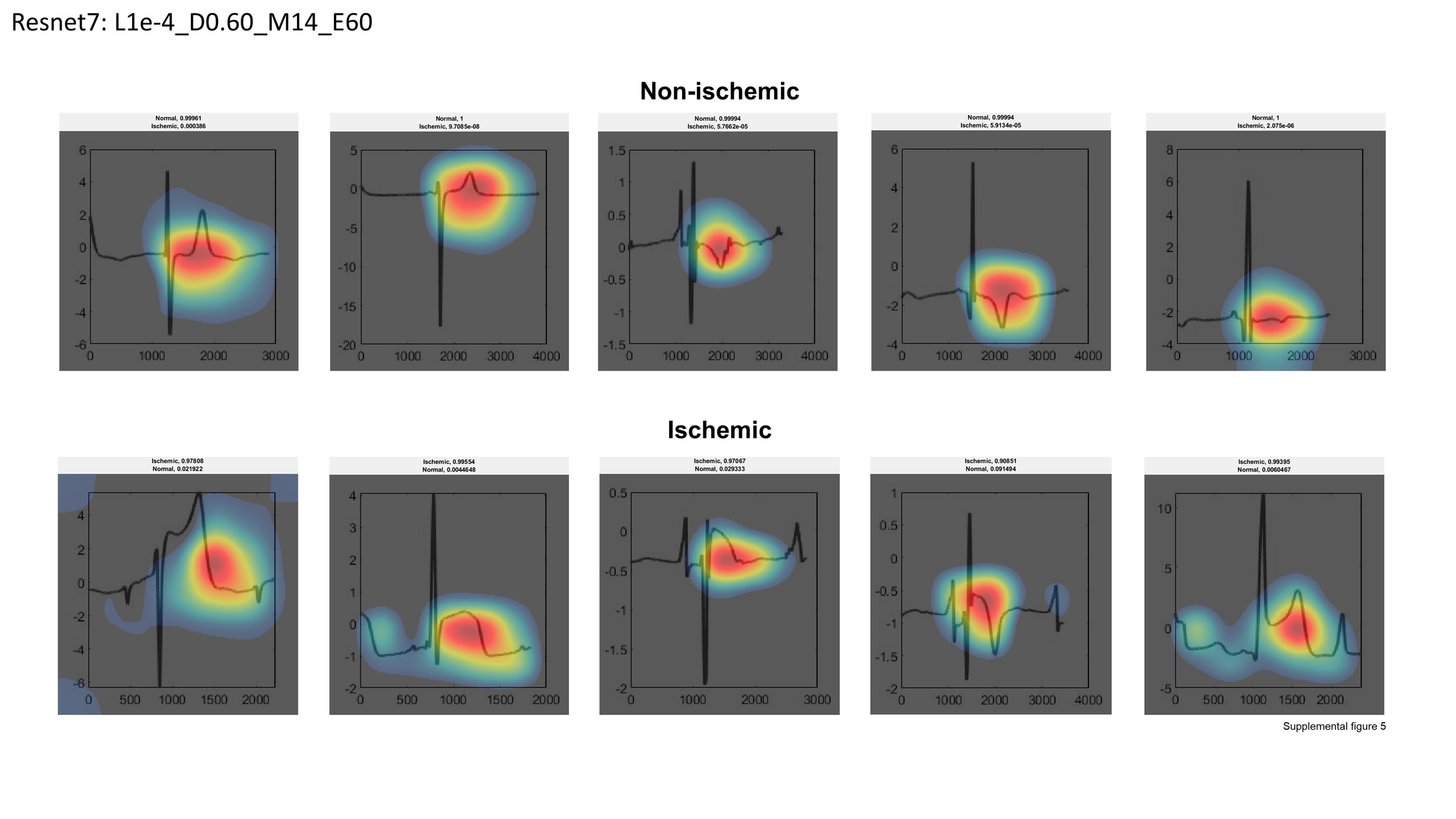

Supplement: S5 Fig — Red regions contributed most to the network class prediction. (TIF) [file pone.0253200.s005.tif]

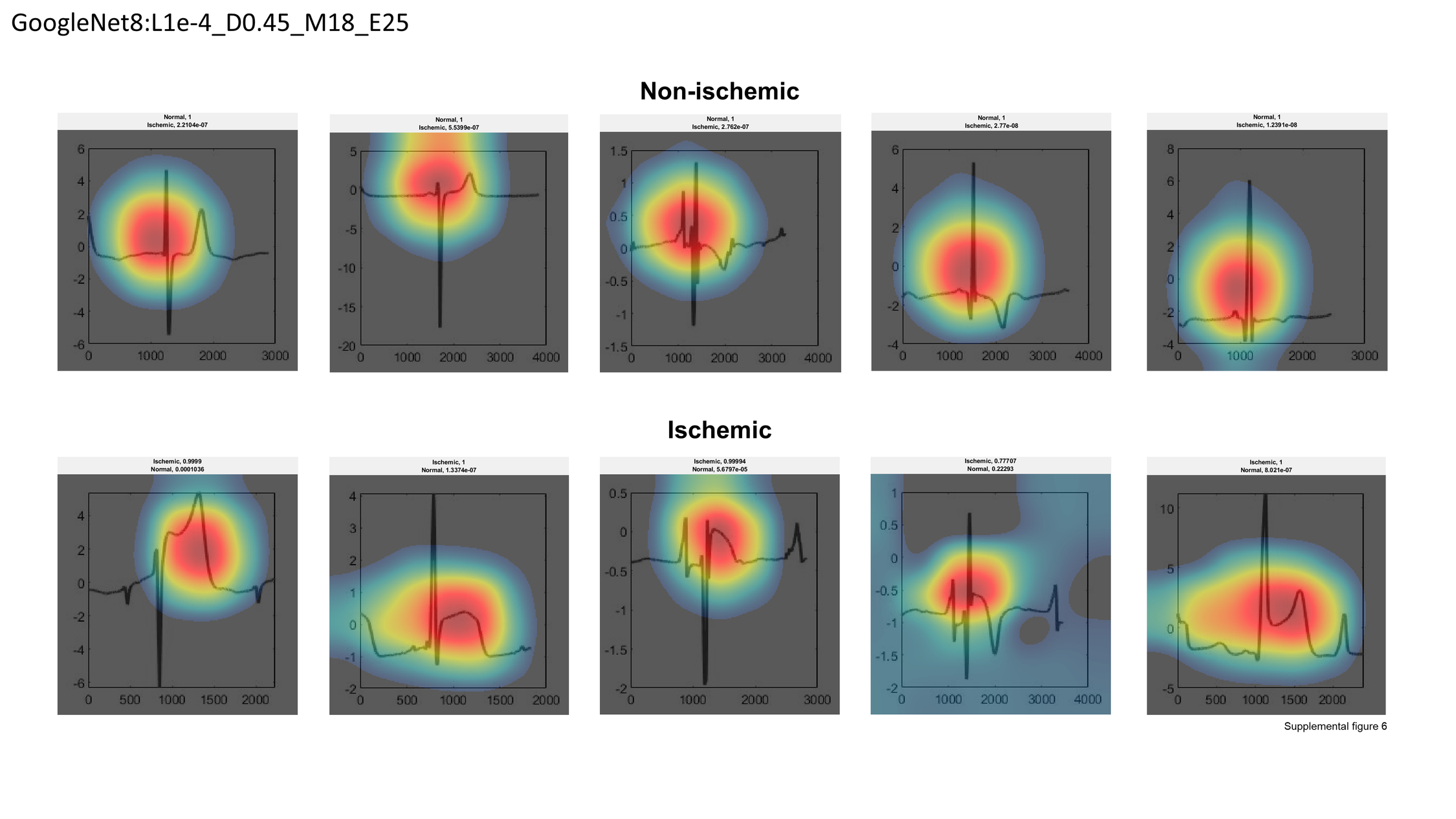

Supplement: S6 Fig — Red regions contributed most to the network class prediction. (TIF) [file pone.0253200.s006.tif]

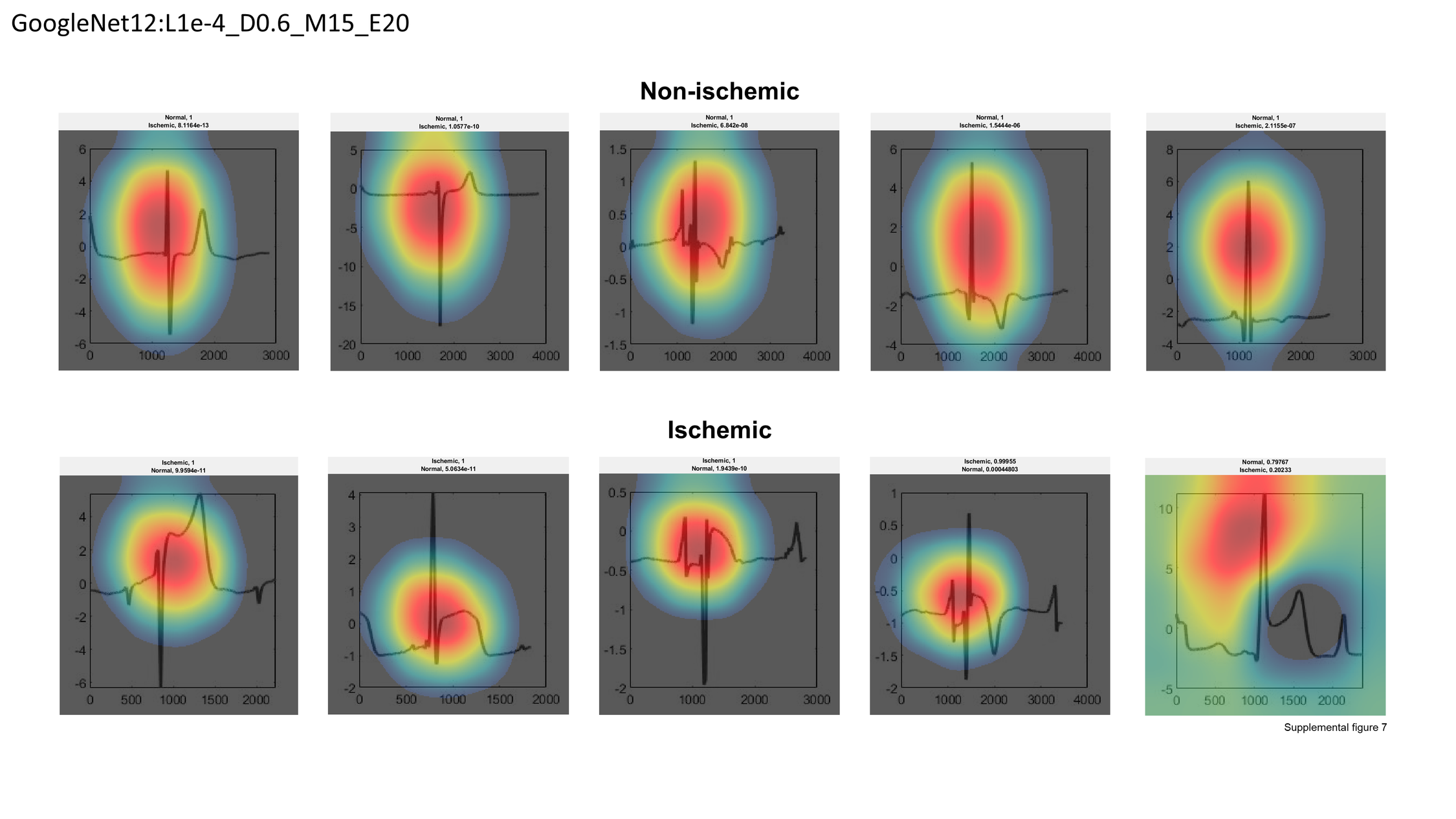

Supplement: S7 Fig — Red regions contributed most to the network class prediction. (TIF) [file pone.0253200.s007.tif]

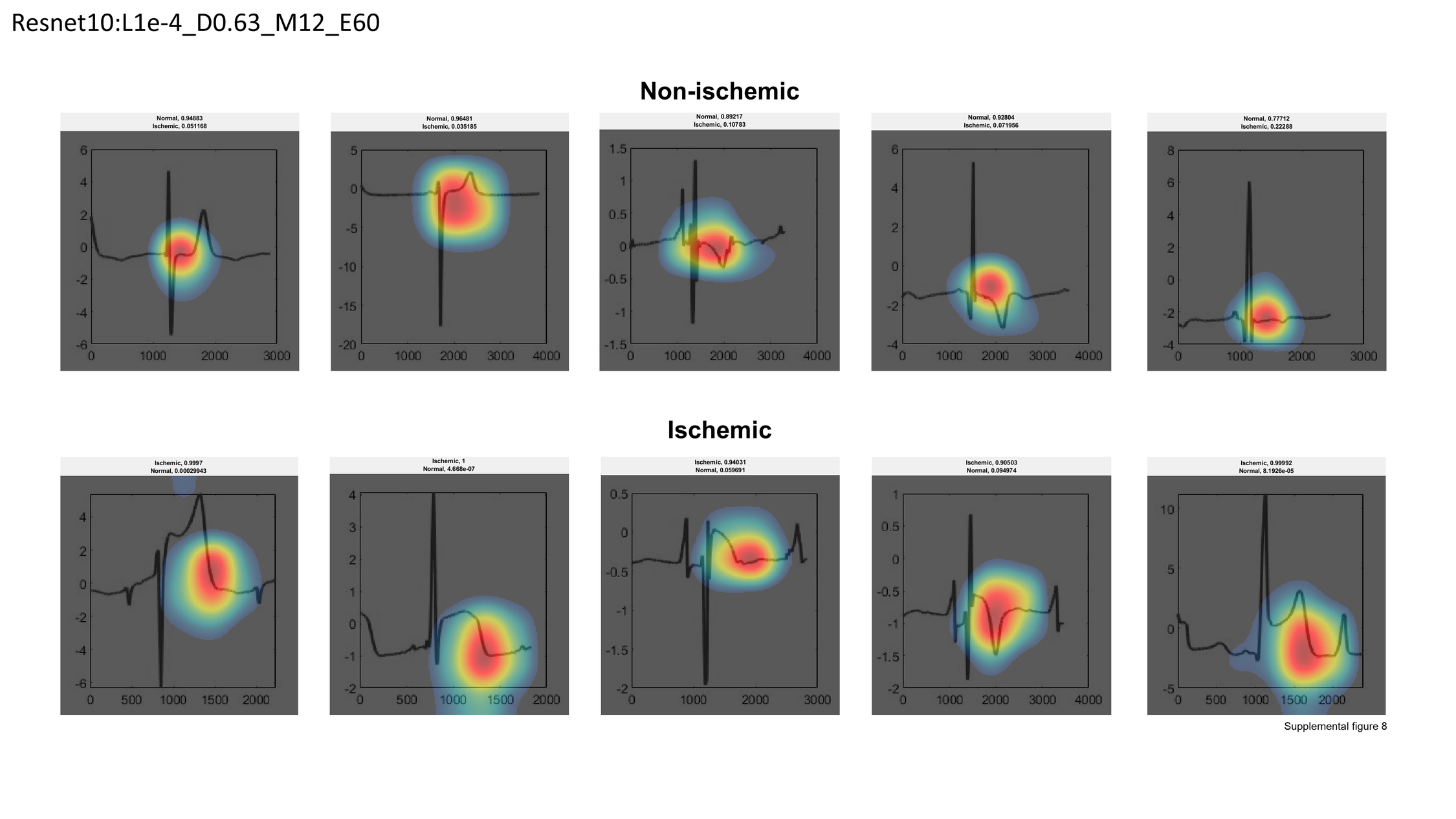

Supplement: S8 Fig — Red regions contributed most to the network class prediction. (TIF) [file pone.0253200.s008.tif]

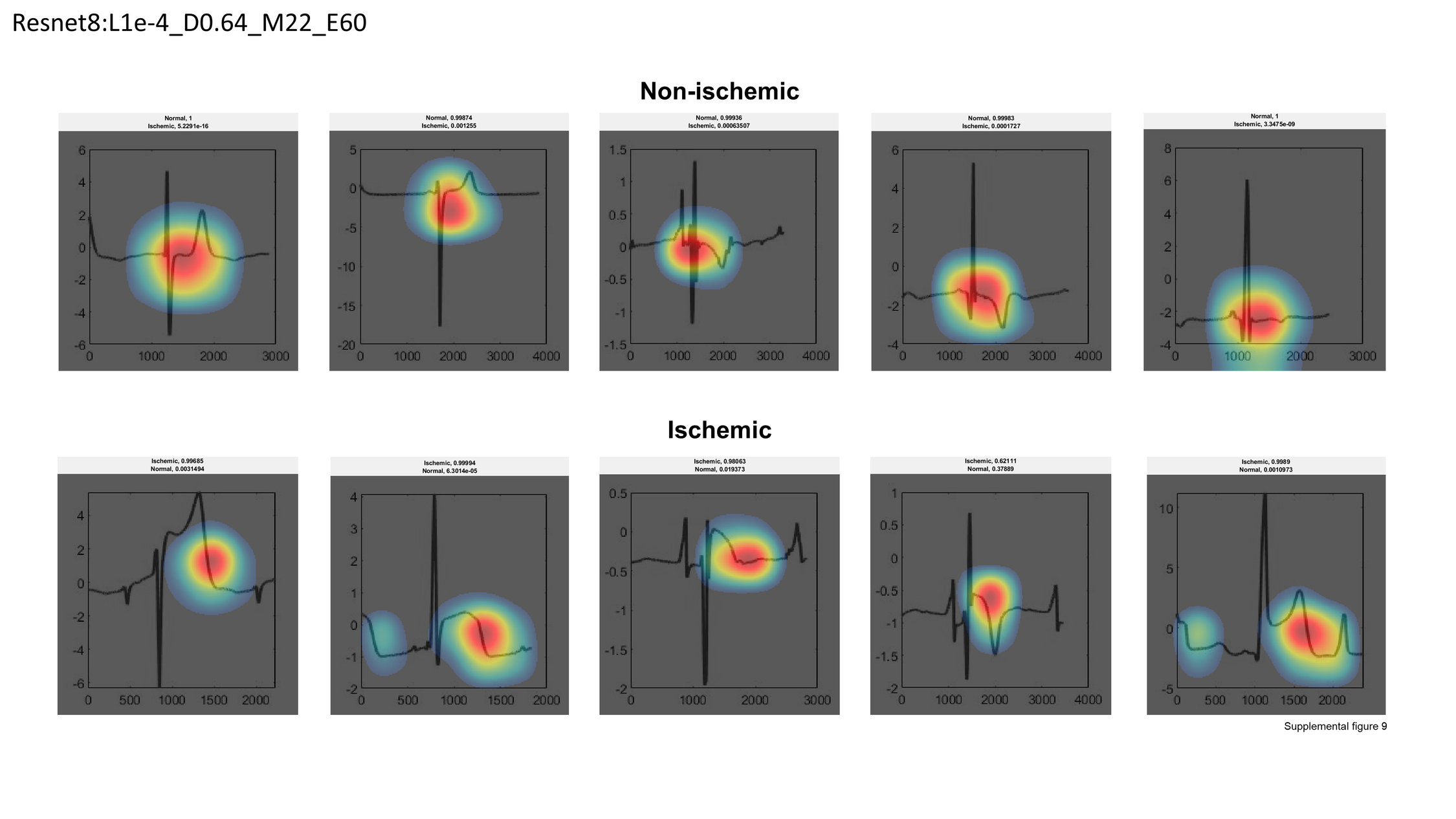

Supplement: S9 Fig — Red regions contributed most to the network class prediction. (TIF) [file pone.0253200.s009.tif]

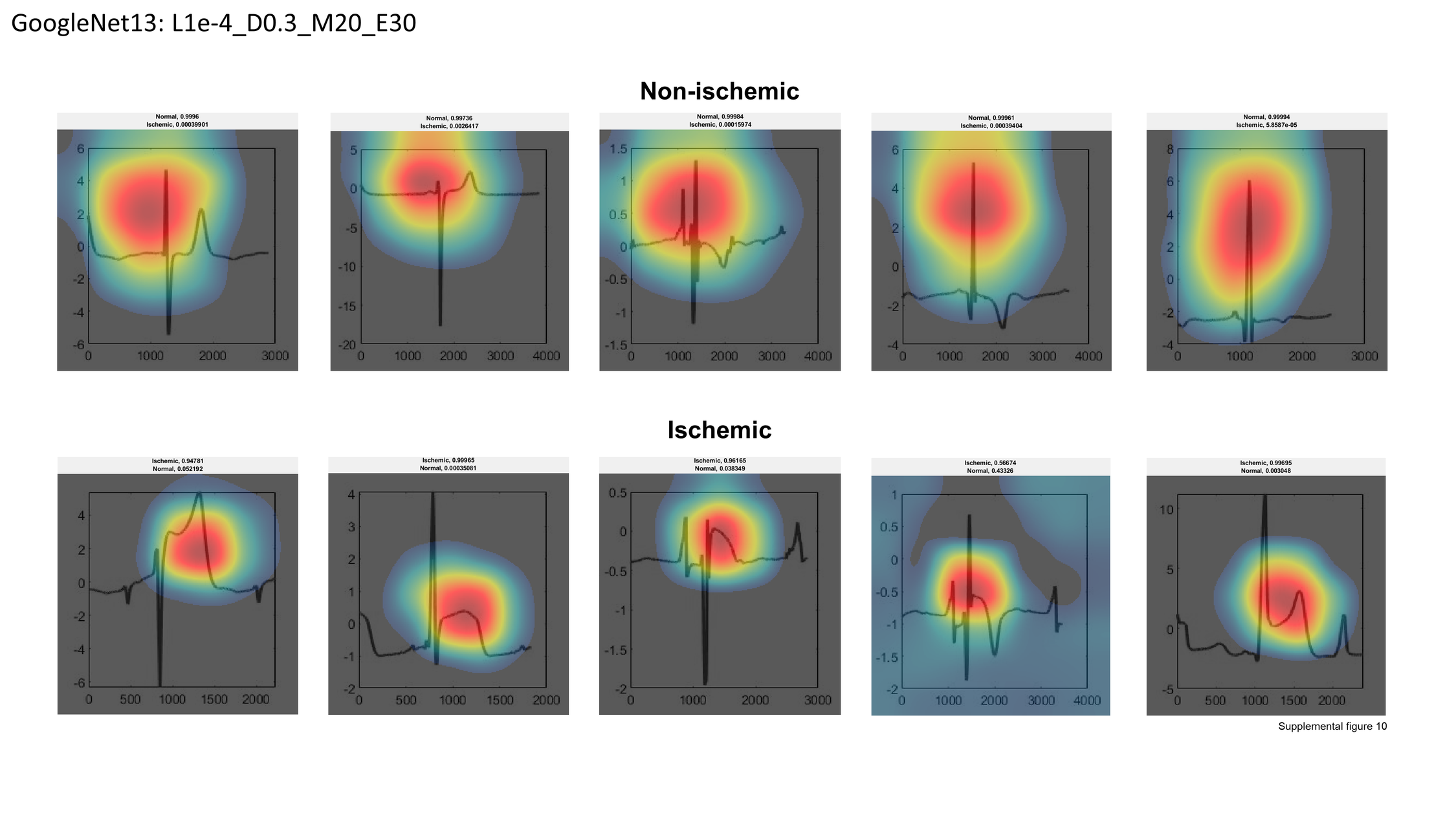

Supplement: S10 Fig — Red regions contributed most to the network class prediction. (TIF) [file pone.0253200.s010.tif]

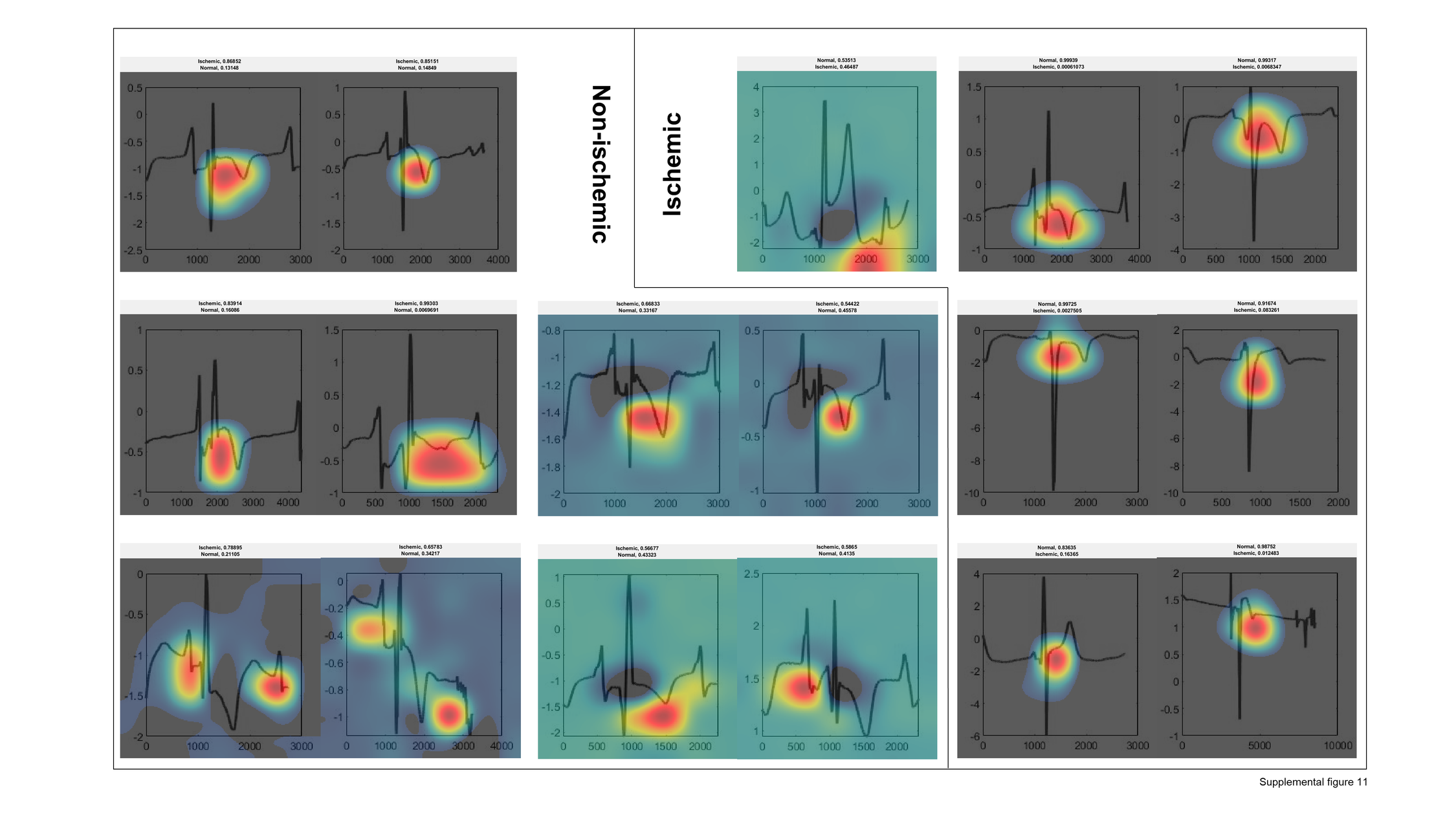

Supplement: S11 Fig — Red regions contributed most to the network class prediction. While some predictions are understandable (left and right column), others are incomprehensible (middle column). (TIF) [file pone.0253200.s011.tif]

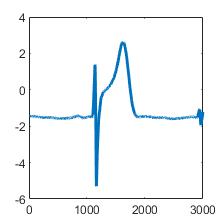

Supplement: S1 Data — (ZIP) [file pone.0253200.s014.zip › Data PlosOne/Ischemic/35002_FU_2_vessel1_arm_1_cfi60_ECGavg.mat.jpg]

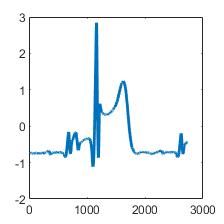

Supplement: S1 Data — (ZIP) [file pone.0253200.s014.zip › Data PlosOne/Ischemic/35002_FU_2_vessel2_arm_1_cfi60_ECGavg.mat.jpg]

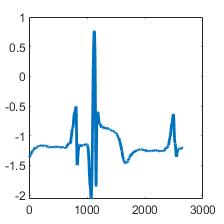

Supplement: S1 Data — (ZIP) [file pone.0253200.s014.zip › Data PlosOne/Ischemic/35002_FU_vessel1_arm_1_cfi60_ECGavg.mat.jpg]

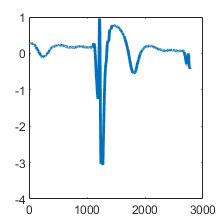

Supplement: S1 Data — (ZIP) [file pone.0253200.s014.zip › Data PlosOne/Ischemic/35003_FU_vessel1_arm_1_cfi60_ECGavg.mat.jpg]

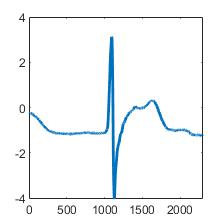

Supplement: S1 Data — (ZIP) [file pone.0253200.s014.zip › Data PlosOne/Ischemic/35003_FU_vessel2_arm_1_cfi60_ECGavg.mat.jpg]

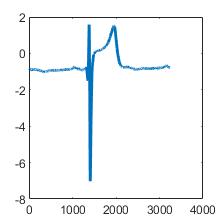

Supplement: S1 Data — (ZIP) [file pone.0253200.s014.zip › Data PlosOne/Ischemic/35004_BL_vessel1_arm_1_cfi60_ECGavg.mat.jpg]

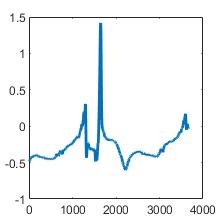

Supplement: S1 Data — (ZIP) [file pone.0253200.s014.zip › Data PlosOne/Ischemic/35004_BL_vessel2_arm_1_cfi60_ECGavg.mat.jpg]

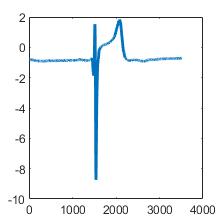

Supplement: S1 Data — (ZIP) [file pone.0253200.s014.zip › Data PlosOne/Ischemic/35004_FU_vessel1_arm_1_cfi60_ECGavg.mat.jpg]

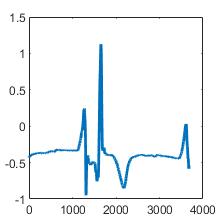

Supplement: S1 Data — (ZIP) [file pone.0253200.s014.zip › Data PlosOne/Ischemic/35004_FU_vessel2_arm_1_cfi60_ECGavg.mat.jpg]

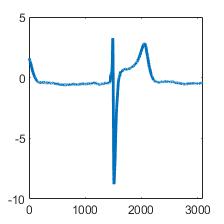

Supplement: S1 Data — (ZIP) [file pone.0253200.s014.zip › Data PlosOne/Ischemic/35005_BL_vessel1_arm_1_cfi60_ECGavg.mat.jpg]

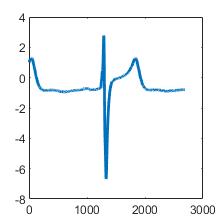

Supplement: S1 Data — (ZIP) [file pone.0253200.s014.zip › Data PlosOne/Ischemic/35005_FU_vessel1_arm_1_cfi60_ECGavg.mat.jpg]

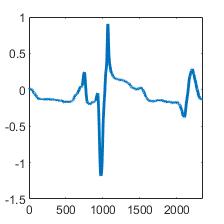

Supplement: S1 Data — (ZIP) [file pone.0253200.s014.zip › Data PlosOne/Ischemic/35006_BL_vessel1_arm_1_cfi60_ECGavg.mat.jpg]

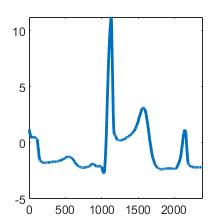

Supplement: S1 Data — (ZIP) [file pone.0253200.s014.zip › Data PlosOne/Ischemic/35006_BL_vessel2_arm_1_cfi60_ECGavg.mat.jpg]

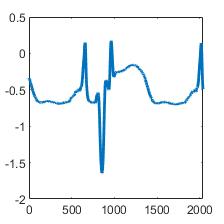

Supplement: S1 Data — (ZIP) [file pone.0253200.s014.zip › Data PlosOne/Ischemic/35006_FU_vessel1_arm_1_cfi60_ECGavg.mat.jpg]

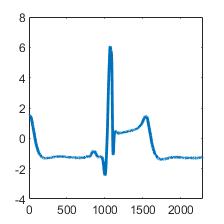

Supplement: S1 Data — (ZIP) [file pone.0253200.s014.zip › Data PlosOne/Ischemic/35006_FU_vessel2_arm_1_cfi60_ECGavg.mat.jpg]

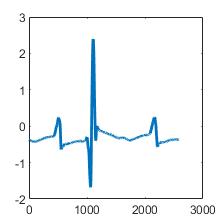

Supplement: S1 Data — (ZIP) [file pone.0253200.s014.zip › Data PlosOne/Ischemic/35007_BL_vessel1_arm_1_cfi60_ECGavg.mat.jpg]

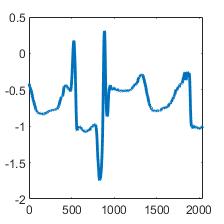

Supplement: S1 Data — (ZIP) [file pone.0253200.s014.zip › Data PlosOne/Ischemic/35008_BL_vessel1_arm_1_cfi60_ECGavg.mat.jpg]

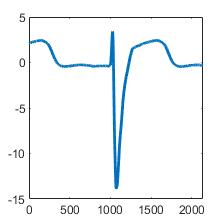

Supplement: S1 Data — (ZIP) [file pone.0253200.s014.zip › Data PlosOne/Ischemic/35009_BL_vessel1_arm_1_cfi60_ECGavg.mat.jpg]

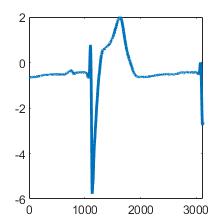

Supplement: S1 Data — (ZIP) [file pone.0253200.s014.zip › Data PlosOne/Ischemic/35009_BL_vessel2_arm_1_cfi60_ECGavg.mat.jpg]

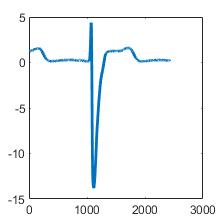

Supplement: S1 Data — (ZIP) [file pone.0253200.s014.zip › Data PlosOne/Ischemic/35009_FU_vessel1_arm_1_cfi60_ECGavg.mat.jpg]

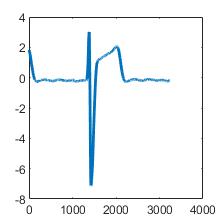

Supplement: S1 Data — (ZIP) [file pone.0253200.s014.zip › Data PlosOne/Ischemic/35009_FU_vessel2_arm_1_cfi60_ECGavg.mat.jpg]

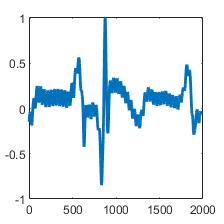

Supplement: S1 Data — (ZIP) [file pone.0253200.s014.zip › Data PlosOne/Ischemic/35010_BL_vessel2_arm_1_cfi60_ECGavg.mat.jpg]

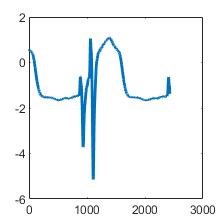

Supplement: S1 Data — (ZIP) [file pone.0253200.s014.zip › Data PlosOne/Ischemic/35010_FU_vessel1_arm_1_cfi60_ECGavg.mat.jpg]

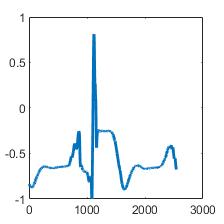

Supplement: S1 Data — (ZIP) [file pone.0253200.s014.zip › Data PlosOne/Ischemic/35010_FU_vessel2_arm_1_cfi60_ECGavg.mat.jpg]

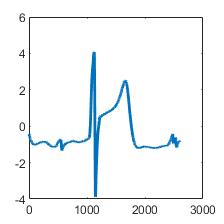

Supplement: S1 Data — (ZIP) [file pone.0253200.s014.zip › Data PlosOne/Ischemic/35011_BL_vessel1_arm_1_cfi60_ECGavg.mat.jpg]

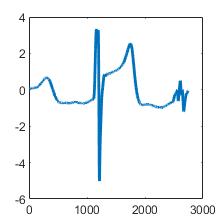

Supplement: S1 Data — (ZIP) [file pone.0253200.s014.zip › Data PlosOne/Ischemic/35011_FU_vessel1_arm_1_cfi60_ECGavg.mat.jpg]

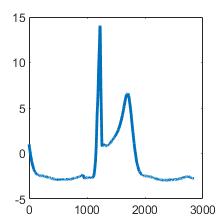

Supplement: S1 Data — (ZIP) [file pone.0253200.s014.zip › Data PlosOne/Ischemic/35012_BL_vessel1_arm_1_cfi60_ECGavg.mat.jpg]

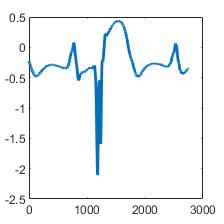

Supplement: S1 Data — (ZIP) [file pone.0253200.s014.zip › Data PlosOne/Ischemic/35012_BL_vessel2_arm_1_cfi60_ECGavg.mat.jpg]

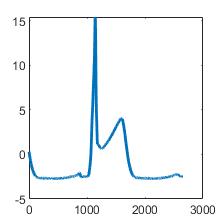

Supplement: S1 Data — (ZIP) [file pone.0253200.s014.zip › Data PlosOne/Ischemic/35012_FU_vessel1_arm_1_cfi60_ECGavg.mat.jpg]

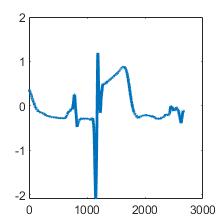

Supplement: S1 Data — (ZIP) [file pone.0253200.s014.zip › Data PlosOne/Ischemic/35012_FU_vessel2_arm_1_cfi60_ECGavg.mat.jpg]

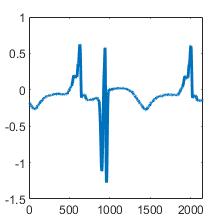

Supplement: S1 Data — (ZIP) [file pone.0253200.s014.zip › Data PlosOne/Ischemic/35013_BL_vessel2_arm_1_cfi60_ECGavg.mat.jpg]

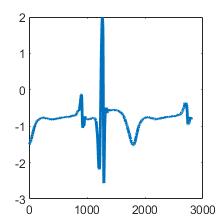

Supplement: S1 Data — (ZIP) [file pone.0253200.s014.zip › Data PlosOne/Ischemic/35014_BL_vessel1_arm_1_cfi60_ECGavg.mat.jpg]

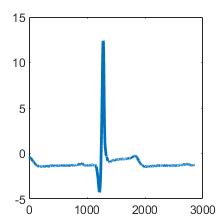

Supplement: S1 Data — (ZIP) [file pone.0253200.s014.zip › Data PlosOne/Ischemic/35014_FU_vessel1_arm_1_cfi60_ECGavg.mat.jpg]

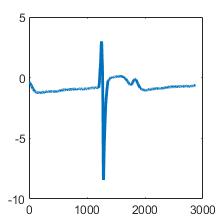

Supplement: S1 Data — (ZIP) [file pone.0253200.s014.zip › Data PlosOne/Ischemic/35015_BL_vessel1_arm_1_cfi60_ECGavg.mat.jpg]

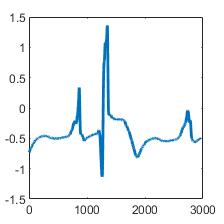

Supplement: S1 Data — (ZIP) [file pone.0253200.s014.zip › Data PlosOne/Ischemic/35015_BL_vessel2_arm_1_cfi60_ECGavg.mat.jpg]

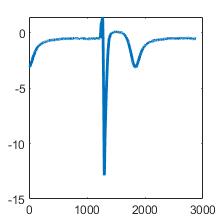

Supplement: S1 Data — (ZIP) [file pone.0253200.s014.zip › Data PlosOne/Ischemic/35015_FU_vessel1_arm_1_cfi60_ECGavg.mat.jpg]

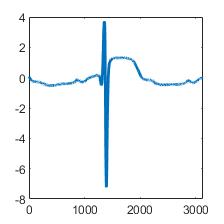

Supplement: S1 Data — (ZIP) [file pone.0253200.s014.zip › Data PlosOne/Ischemic/35015_FU_vessel2_arm_1_cfi60_ECGavg.mat.jpg]

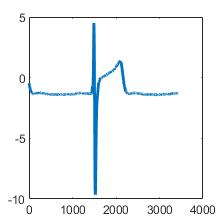

Supplement: S1 Data — (ZIP) [file pone.0253200.s014.zip › Data PlosOne/Ischemic/35016_FU_vessel1_arm_1_cfi60_ECGavg.mat.jpg]

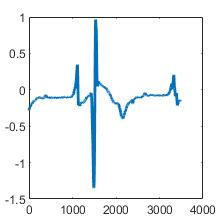

Supplement: S1 Data — (ZIP) [file pone.0253200.s014.zip › Data PlosOne/Ischemic/35016_FU_vessel2_arm_1_cfi60_ECGavg.mat.jpg]

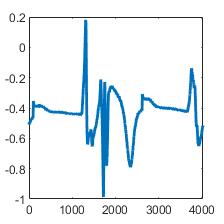

Supplement: S1 Data — (ZIP) [file pone.0253200.s014.zip › Data PlosOne/Ischemic/35017_BL_vessel2_arm_1_cfi60_ECGavg.mat.jpg]

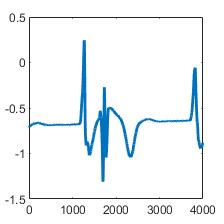

Supplement: S1 Data — (ZIP) [file pone.0253200.s014.zip › Data PlosOne/Ischemic/35017_FU_vessel1_arm_1_cfi60_ECGavg.mat.jpg]

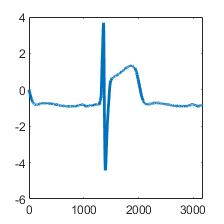

Supplement: S1 Data — (ZIP) [file pone.0253200.s014.zip › Data PlosOne/Ischemic/35017_FU_vessel2_arm_1_cfi60_ECGavg.mat.jpg]

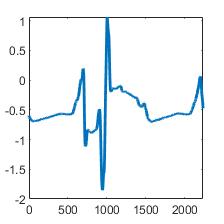

Supplement: S1 Data — (ZIP) [file pone.0253200.s014.zip › Data PlosOne/Ischemic/35018_BL_vessel1_arm_1_cfi60_ECGavg.mat.jpg]

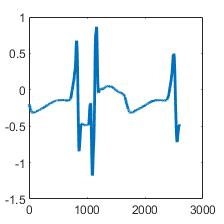

Supplement: S1 Data — (ZIP) [file pone.0253200.s014.zip › Data PlosOne/Ischemic/35018_FU_vessel1_arm_1_cfi60_ECGavg.mat.jpg]

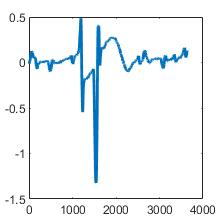

Supplement: S1 Data — (ZIP) [file pone.0253200.s014.zip › Data PlosOne/Ischemic/35019_BL_vessel1_arm_1_cfi60_ECGavg.mat.jpg]

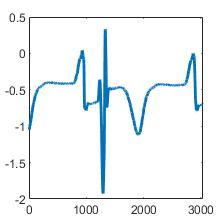

Supplement: S1 Data — (ZIP) [file pone.0253200.s014.zip › Data PlosOne/Ischemic/35019_FU_vessel1_arm_1_cfi60_ECGavg.mat.jpg]

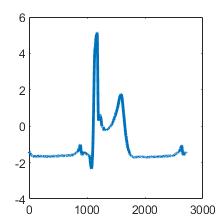

Supplement: S1 Data — (ZIP) [file pone.0253200.s014.zip › Data PlosOne/Ischemic/35020_BL_vessel1_arm_1_cfi60_ECGavg.mat.jpg]

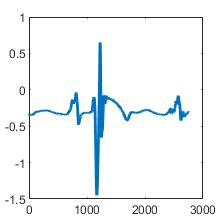

Supplement: S1 Data — (ZIP) [file pone.0253200.s014.zip › Data PlosOne/Ischemic/35020_BL_vessel2_arm_1_cfi60_ECGavg.mat.jpg]

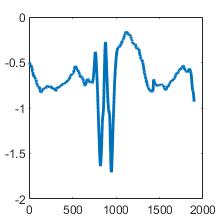

Supplement: S1 Data — (ZIP) [file pone.0253200.s014.zip › Data PlosOne/Ischemic/35021_BL_vessel1_arm_1_cfi60_ECGavg.mat.jpg]

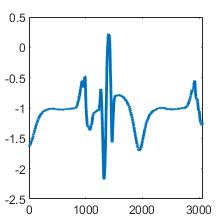

Supplement: S1 Data — (ZIP) [file pone.0253200.s014.zip › Data PlosOne/Ischemic/35021_FU_vessel1_arm_1_cfi60_ECGavg.mat.jpg]

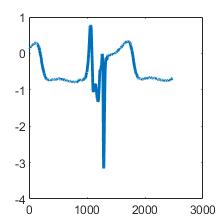

Supplement: S1 Data — (ZIP) [file pone.0253200.s014.zip › Data PlosOne/Ischemic/35022_BL_vessel1_arm_1_cfi60_ECGavg.mat.jpg]

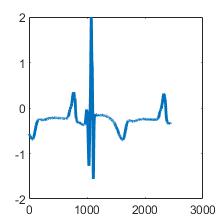

Supplement: S1 Data — (ZIP) [file pone.0253200.s014.zip › Data PlosOne/Ischemic/35022_BL_vessel2_arm_1_cfi60_ECGavg.mat.jpg]

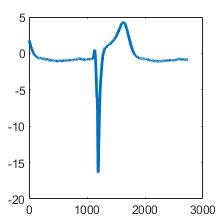

Supplement: S1 Data — (ZIP) [file pone.0253200.s014.zip › Data PlosOne/Ischemic/35023_BL_vessel1_arm_1_cfi60_ECGavg.mat.jpg]

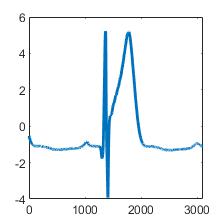

Supplement: S1 Data — (ZIP) [file pone.0253200.s014.zip › Data PlosOne/Ischemic/35023_BL_vessel2_arm_1_cfi60_ECGavg.mat.jpg]

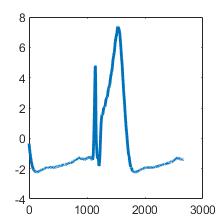

Supplement: S1 Data — (ZIP) [file pone.0253200.s014.zip › Data PlosOne/Ischemic/35023_FU_vessel1_arm_1_cfi60_ECGavg.mat.jpg]

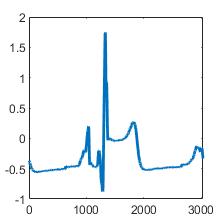

Supplement: S1 Data — (ZIP) [file pone.0253200.s014.zip › Data PlosOne/Ischemic/35024_FU_vessel1_arm_1_cfi60_ECGavg.mat.jpg]

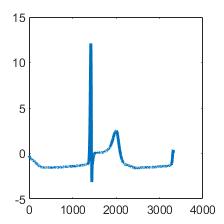

Supplement: S1 Data — (ZIP) [file pone.0253200.s014.zip › Data PlosOne/Ischemic/35025_BL_vessel1_arm_1_cfi60_ECGavg.mat.jpg]

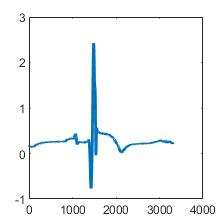

Supplement: S1 Data — (ZIP) [file pone.0253200.s014.zip › Data PlosOne/Ischemic/35025_BL_vessel2_arm_1_cfi60_ECGavg.mat.jpg]

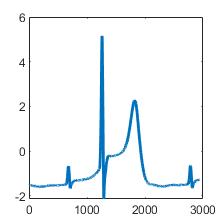

Supplement: S1 Data — (ZIP) [file pone.0253200.s014.zip › Data PlosOne/Ischemic/35025_FU_vessel1_arm_1_cfi60_ECGavg.mat.jpg]

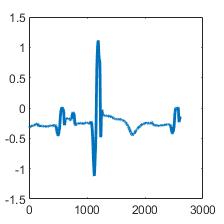

Supplement: S1 Data — (ZIP) [file pone.0253200.s014.zip › Data PlosOne/Ischemic/35025_FU_vessel3_arm_1_cfi60_ECGavg.mat.jpg]

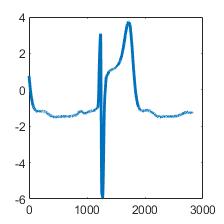

Supplement: S1 Data — (ZIP) [file pone.0253200.s014.zip › Data PlosOne/Ischemic/35026_BL_vessel1_arm_1_cfi60_ECGavg.mat.jpg]

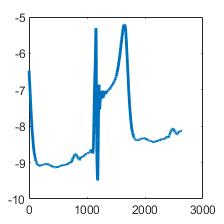

Supplement: S1 Data — (ZIP) [file pone.0253200.s014.zip › Data PlosOne/Ischemic/35026_FU_vessel1_arm_1_cfi60_ECGavg.mat.jpg]

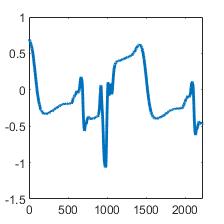

Supplement: S1 Data — (ZIP) [file pone.0253200.s014.zip › Data PlosOne/Ischemic/35027_BL_vessel1_arm_1_cfi60_ECGavg.mat.jpg]

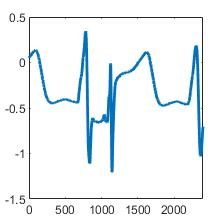

Supplement: S1 Data — (ZIP) [file pone.0253200.s014.zip › Data PlosOne/Ischemic/35028_BL_vessel1_arm_1_cfi60_ECGavg.mat.jpg]

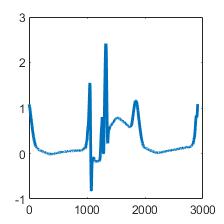

Supplement: S1 Data — (ZIP) [file pone.0253200.s014.zip › Data PlosOne/Ischemic/35028_BL_vessel2_arm_1_cfi60_ECGavg.mat.jpg]

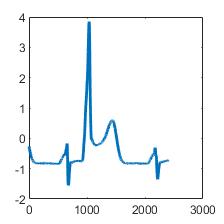

Supplement: S1 Data — (ZIP) [file pone.0253200.s014.zip › Data PlosOne/Ischemic/35028_FU_vessel1_arm_1_cfi60_ECGavg.mat.jpg]

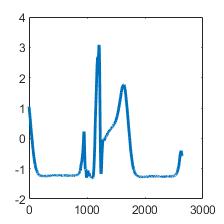

Supplement: S1 Data — (ZIP) [file pone.0253200.s014.zip › Data PlosOne/Ischemic/35028_FU_vessel2_arm_1_cfi60_ECGavg.mat.jpg]

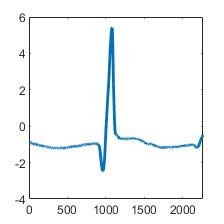

Supplement: S1 Data — (ZIP) [file pone.0253200.s014.zip › Data PlosOne/Ischemic/35029_FU_vessel2_arm_1_cfi60_ECGavg.mat.jpg]

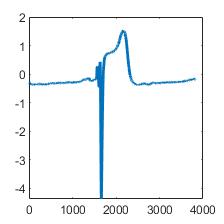

Supplement: S1 Data — (ZIP) [file pone.0253200.s014.zip › Data PlosOne/Ischemic/35030_BL_vessel1_arm_1_cfi60_ECGavg.mat.jpg]

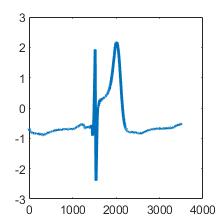

Supplement: S1 Data — (ZIP) [file pone.0253200.s014.zip › Data PlosOne/Ischemic/35030_FU_vessel1_arm_1_cfi60_ECGavg.mat.jpg]

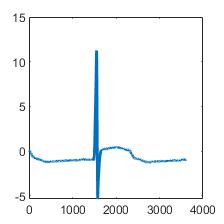

Supplement: S1 Data — (ZIP) [file pone.0253200.s014.zip › Data PlosOne/Ischemic/35031_BL_vessel1_arm_1_cfi60_ECGavg.mat.jpg]

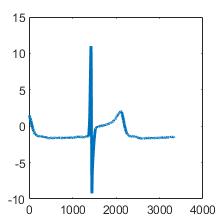

Supplement: S1 Data — (ZIP) [file pone.0253200.s014.zip › Data PlosOne/Ischemic/35031_FU_vessel1_arm_1_cfi60_ECGavg.mat.jpg]

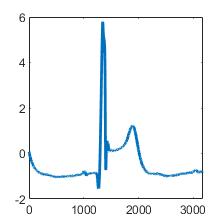

Supplement: S1 Data — (ZIP) [file pone.0253200.s014.zip › Data PlosOne/Ischemic/35032_BL_vessel1_arm_1_cfi60_ECGavg.mat.jpg]

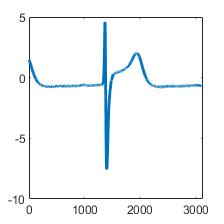

Supplement: S1 Data — (ZIP) [file pone.0253200.s014.zip › Data PlosOne/Ischemic/35032_BL_vessel2_arm_1_cfi60_ECGavg.mat.jpg]

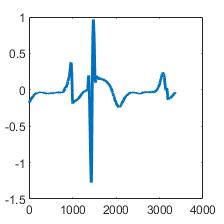

Supplement: S1 Data — (ZIP) [file pone.0253200.s014.zip › Data PlosOne/Ischemic/35032_BL_vessel3_arm_1_cfi60_ECGavg.mat.jpg]

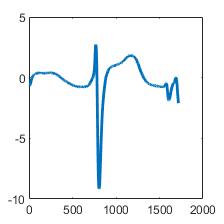

Supplement: S1 Data — (ZIP) [file pone.0253200.s014.zip › Data PlosOne/Ischemic/35032_FU_2_vessel2_arm_1_cfi60_ECGavg.mat.jpg]

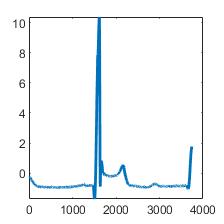

Supplement: S1 Data — (ZIP) [file pone.0253200.s014.zip › Data PlosOne/Ischemic/35032_FU_vessel1_arm_1_cfi60_ECGavg.mat.jpg]

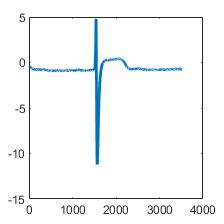

Supplement: S1 Data — (ZIP) [file pone.0253200.s014.zip › Data PlosOne/Ischemic/35032_FU_vessel2_arm_1_cfi60_ECGavg.mat.jpg]

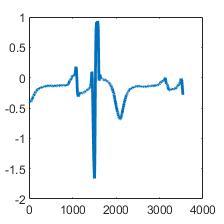

Supplement: S1 Data — (ZIP) [file pone.0253200.s014.zip › Data PlosOne/Ischemic/35032_FU_vessel4_arm_1_cfi60_ECGavg.mat.jpg]

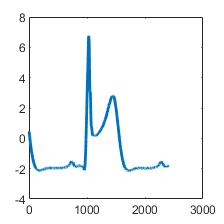

Supplement: S1 Data — (ZIP) [file pone.0253200.s014.zip › Data PlosOne/Ischemic/35033_BL_vessel2_arm_1_cfi60_ECGavg.mat.jpg]

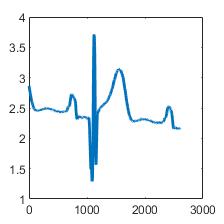

Supplement: S1 Data — (ZIP) [file pone.0253200.s014.zip › Data PlosOne/Ischemic/35033_FU_vessel1_arm_1_cfi60_ECGavg.mat.jpg]

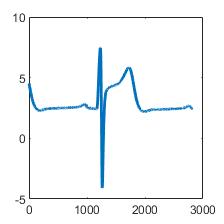

Supplement: S1 Data — (ZIP) [file pone.0253200.s014.zip › Data PlosOne/Ischemic/35033_FU_vessel2_arm_1_cfi60_ECGavg.mat.jpg]

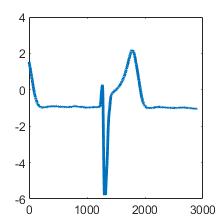

Supplement: S1 Data — (ZIP) [file pone.0253200.s014.zip › Data PlosOne/Ischemic/35034_BL_vessel1_arm_1_cfi60_ECGavg.mat.jpg]

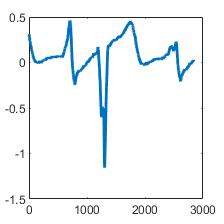

Supplement: S1 Data — (ZIP) [file pone.0253200.s014.zip › Data PlosOne/Ischemic/35034_BL_vessel2_arm_1_cfi60_ECGavg.mat.jpg]

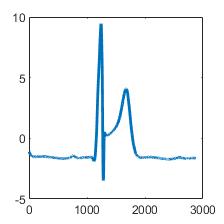

Supplement: S1 Data — (ZIP) [file pone.0253200.s014.zip › Data PlosOne/Ischemic/35034_BL_vessel3_arm_1_cfi60_ECGavg.mat.jpg]

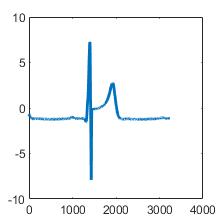

Supplement: S1 Data — (ZIP) [file pone.0253200.s014.zip › Data PlosOne/Ischemic/35034_FU_vessel1_arm_1_cfi60_ECGavg.mat.jpg]

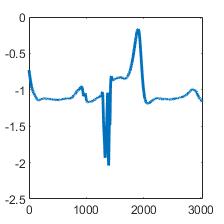

Supplement: S1 Data — (ZIP) [file pone.0253200.s014.zip › Data PlosOne/Ischemic/35034_FU_vessel2_arm_1_cfi60_ECGavg.mat.jpg]

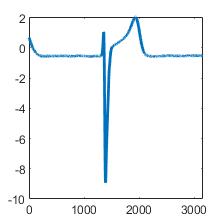

Supplement: S1 Data — (ZIP) [file pone.0253200.s014.zip › Data PlosOne/Ischemic/35034_FU_vessel3_arm_1_cfi60_ECGavg.mat.jpg]

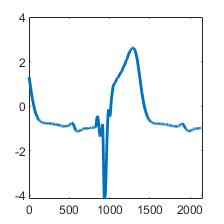

Supplement: S1 Data — (ZIP) [file pone.0253200.s014.zip › Data PlosOne/Ischemic/35035_BL_vessel1_arm_1_cfi60_ECGavg.mat.jpg]
