# Supplementary material for: Detection of myocardial ischemia by intracoronary ECG using convolutional neural networks
Source: PLoS One. 2021 Jun 14;16(6):e0253200. doi: 10.1371/journal.pone.0253200 (PMC8202932; doi:10.1371/journal.pone.0253200)
Supplement: S1 Table — (DOCX) [file pone.0253200.s012.docx]

S1 Table: Nonparametric receiver-operating characteristic curves and corresponding collateral flow index and heart rate

|  | **Overall** |
| --- | --- |
| **Overall, n** | **893** |
| AUC | 0.932±0.018 |
| CFI (mmHg/mmHg) | 0.116±0.085 |
| Heart rate (bpm) | 73±14 |
| **Training data, n** | **668** |
| AUC | 0.941±0.019 |
| CFI (mmHg/mmHg) | 0.114±0.086 |
| Heart rate (bpm) | 74±14 |
| **Validation data, n** | **167** |
| AUC | 0.897±0.051 |
| CFI (mmHg/mmHg) | 0.122±0.080 |
| Heart rate (bpm) | 72±14 |
| **Examination data, n** | **58** |
| AUC | 0.927±0.074 |
| CFI (mmHg/mmHg) | 0.126±0.084 |
| Heart rate (bpm) | 74±16 |
| **Validation + Examination data, n** | **225** |
| AUC | 0.903±0.043 |
| CFI (mmHg/mmHg) | 0.123±0.081 |
| Heart rate (bpm) | 72±14 |
| AUC = Area under the ROC-curve; CFI = Collateral flow index | |
