# Supplementary material for: Detection of myocardial ischemia by intracoronary ECG using convolutional neural networks
Source: PLoS One. 2021 Jun 14;16(6):e0253200. doi: 10.1371/journal.pone.0253200 (PMC8202932; doi:10.1371/journal.pone.0253200)
Supplement: S2 Table — (DOCX) [file pone.0253200.s013.docx]

|  | **Training Data** | | | | **Validation Data** | | | | **Examination Data** | | | | |
| --- | --- | --- | --- | --- | --- | --- | --- | --- | --- | --- | --- | --- | --- |
| **Convolutional neural network** | True  Predicted | **Non-Ischemic** | **Ischemic** | **Accuracy** | True  Predicted | **Non-Ischemic** | **Ischemic** | **Accuracy** | True  Predicted | **Non-Ischemic** | **Ischemic** | **Accuracy** |  |
| **ResNet5:**  L1e-4_D0.80_M14_E60 | Non-Ischemic | 350 | 10 | 95.81 | Non-Ischemic | 88 | 6 | 94.01 | Non-Ischemic | 25 | 1 | 87.93 |  |
|  | Ischemic | 18 | 290 |  | Ischemic | 4 | 69 |  | Ischemic | 6 | 26 |  |  |
| **GoogLeNet10:**  L1e-4_D0.26_M18_E28 | Non-Ischemic | 363 | 4 | 98.65 | Non-Ischemic | 88 | 5 | 94.61 | Non-Ischemic | 29 | 6 | 86.21 |  |
|  | Ischemic | 5 | 296 |  | Ischemic | 4 | 70 |  | Ischemic | 2 | 21 |  |  |
| **ResNet6:**  L2e-4_D0.52_M25_E60 | Non-Ischemic | 363 | 15 | 97.01 | Non-Ischemic | 90 | 10 | 92.81 | Non-Ischemic | 28 | 2 | 91.38 |  |
|  | Ischemic | 5 | 285 |  | Ischemic | 2 | 65 |  | Ischemic | 3 | 25 |  |  |
| **GoogLeNet2:**  L1e-4_D0.25_M18_E20 | Non-Ischemic | 360 | 9 | 97.46 | Non-Ischemic | 85 | 7 | 91.62 | Non-Ischemic | 29 | 2 | 93.10 |  |
|  | Ischemic | 8 | 291 |  | Ischemic | 7 | 68 |  | Ischemic | 2 | 25 |  |  |
| **ResNet7:**  L1e-4_D0.60_M14_E60 | Non-Ischemic | 351 | 14 | 95.36 | Non-Ischemic | 87 | 8 | 92.22 | Non-Ischemic | 29 | 3 | 91.38 |  |
|  | Ischemic | 17 | 286 |  | Ischemic | 5 | 67 |  | Ischemic | 2 | 24 |  |  |
| **GoogLeNet8:**  L1e-4_D0.45_M18_E25 | Non-Ischemic | 364 | 3 | 98.95 | Non-Ischemic | 89 | 9 | 92.81 | Non-Ischemic | 30 | 5 | 89.66 |  |
|  | Ischemic | 4 | 297 |  | Ischemic | 3 | 66 |  | Ischemic | 1 | 22 |  |  |
| **GoogLeNet12:**  L1e-4_D0.6_M15_E20 | Non-Ischemic | 357 | 47 | 91.32 | Non-Ischemic | 90 | 11 | 92.22 | Non-Ischemic | 30 | 4 | 91.38 |  |
|  | Ischemic | 11 | 253 |  | Ischemic | 2 | 64 |  | Ischemic | 1 | 23 |  |  |
| **ResNet10:**  L1e-4_D0.63_M12_E60 | Non-Ischemic | 352 | 25 | 93.86 | Non-Ischemic | 84 | 8 | 90.42 | Non-Ischemic | 29 | 1 | 94.83 |  |
|  | Ischemic | 16 | 275 |  | Ischemic | 8 | 67 |  | Ischemic | 2 | 26 |  |  |
| **ResNet8:**  L1e-4_D0.64_M22_E60 | Non-Ischemic | 362 | 28 | 94.91 | Non-Ischemic | 90 | 12 | 91.62 | Non-Ischemic | 31 | 5 | 91.38 |  |
|  | Ischemic | 6 | 272 |  | Ischemic | 2 | 63 |  | Ischemic | 0 | 22 |  |  |
| **GoogLeNet13:**  L1e-4_D0.3_M20_E30 | Non-Ischemic | 366 | 9 | 98.35 | Non-Ischemic | 91 | 10 | 93.41 | Non-Ischemic | 30 | 7 | 86.21 |  |
|  | Ischemic | 2 | 291 |  | Ischemic | 1 | 65 |  | Ischemic | 1 | 20 |  |  |
| Order according to accuracy. L = learning rate, D = dropout rate, M = minibatch size, E = number of epochs.  Please note that all ResNet-networks were trained with a preliminary termination term (thus, all had E60 but were automatically stopped by the training algorithm). | | | | | | | | | | | | |  |

S2 Table: In-detail performance of the best ten trained networks
