# Supplementary figures and images for: Detection of myocardial ischemia by intracoronary ECG using convolutional neural networks (part 3 of 3)
Source: PLoS One. 2021 Jun 14;16(6):e0253200. doi: 10.1371/journal.pone.0253200 (PMC8202932; doi:10.1371/journal.pone.0253200)

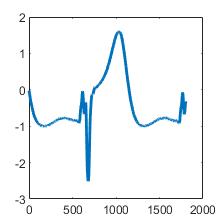

Supplement: S1 Data — (ZIP) [file pone.0253200.s014.zip › Data PlosOne/Ischemic/35080_FU2_vessel2_arm_1_cfi60_ECGavg.mat.jpg]

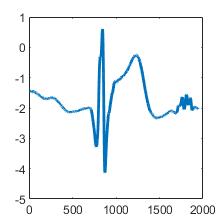

Supplement: S1 Data — (ZIP) [file pone.0253200.s014.zip › Data PlosOne/Ischemic/35081_FU_vessel1_arm_1_cfi60_ECGavg.mat.jpg]

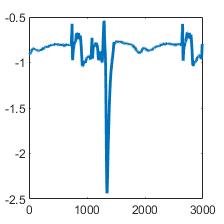

Supplement: S1 Data — (ZIP) [file pone.0253200.s014.zip › Data PlosOne/Ischemic/35082_BL_vessel1_arm_1_cfi60_ECGavg.mat.jpg]

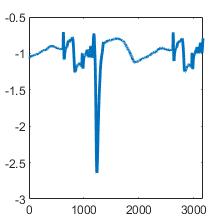

Supplement: S1 Data — (ZIP) [file pone.0253200.s014.zip › Data PlosOne/Ischemic/35082_FU_vessel1_arm_1_cfi60_ECGavg.mat.jpg]

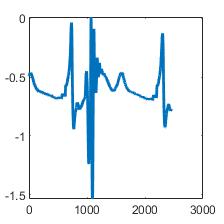

Supplement: S1 Data — (ZIP) [file pone.0253200.s014.zip › Data PlosOne/Ischemic/35083_BL_vessel1_arm_1_cfi60_ECGavg.mat.jpg]

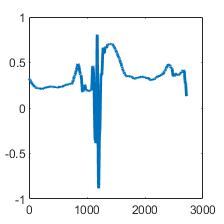

Supplement: S1 Data — (ZIP) [file pone.0253200.s014.zip › Data PlosOne/Ischemic/35083_FU_vessel1_arm_1_cfi60_ECGavg.mat.jpg]

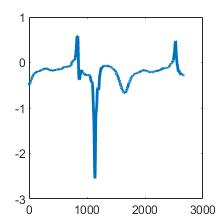

Supplement: S1 Data — (ZIP) [file pone.0253200.s014.zip › Data PlosOne/Ischemic/35084_BL_vessel1_arm_1_cfi60_ECGavg.mat.jpg]

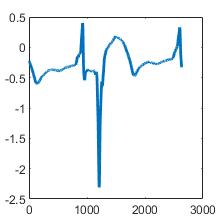

Supplement: S1 Data — (ZIP) [file pone.0253200.s014.zip › Data PlosOne/Ischemic/35084_FU_vessel1_arm_1_cfi60_ECGavg.mat.jpg]

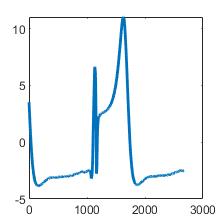

Supplement: S1 Data — (ZIP) [file pone.0253200.s014.zip › Data PlosOne/Ischemic/35084_FU_vessel2_arm_1_cfi60_ECGavg.mat.jpg]

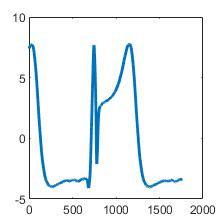

Supplement: S1 Data — (ZIP) [file pone.0253200.s014.zip › Data PlosOne/Ischemic/35084_FU_vessel3_arm_1_cfi60_ECGavg.mat.jpg]

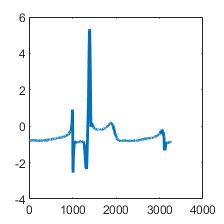

Supplement: S1 Data — (ZIP) [file pone.0253200.s014.zip › Data PlosOne/Ischemic/35085_BL_vessel1_arm_1_cfi60_ECGavg.mat.jpg]

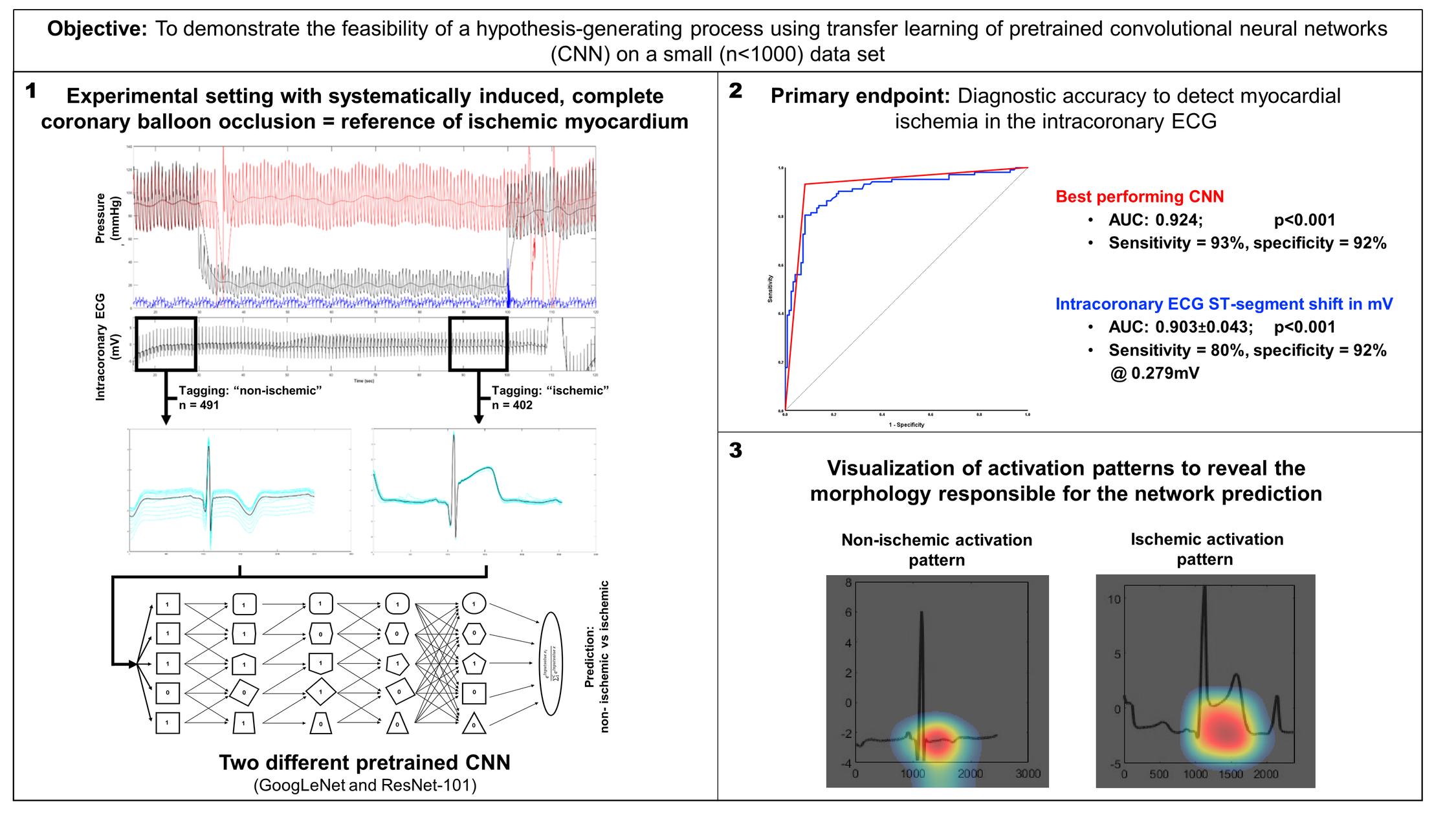

Supplement: S1 Graphical abstract — (TIF) [file pone.0253200.s015.tif]
