# Supplementary figures and images for: Detection of myocardial ischemia by intracoronary ECG using convolutional neural networks (part 2 of 3)
Source: PLoS One. 2021 Jun 14;16(6):e0253200. doi: 10.1371/journal.pone.0253200 (PMC8202932; doi:10.1371/journal.pone.0253200)

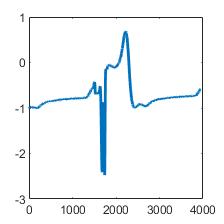

Supplement: S1 Data — (ZIP) [file pone.0253200.s014.zip › Data PlosOne/Ischemic/35036_BL_vessel1_arm_1_cfi60_ECGavg.mat.jpg]

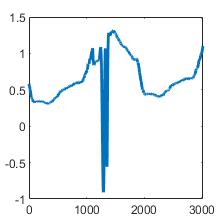

Supplement: S1 Data — (ZIP) [file pone.0253200.s014.zip › Data PlosOne/Ischemic/35036_FU_vessel1_arm_1_cfi60_ECGavg.mat.jpg]

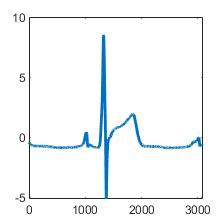

Supplement: S1 Data — (ZIP) [file pone.0253200.s014.zip › Data PlosOne/Ischemic/35037_BL_vessel1_arm_1_cfi60_ECGavg.mat.jpg]

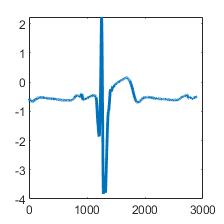

Supplement: S1 Data — (ZIP) [file pone.0253200.s014.zip › Data PlosOne/Ischemic/35037_BL_vessel2_arm_1_cfi60_ECGavg.mat.jpg]

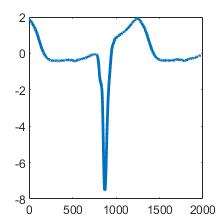

Supplement: S1 Data — (ZIP) [file pone.0253200.s014.zip › Data PlosOne/Ischemic/35037_FU_vessel3_arm_1_cfi60_ECGavg.mat.jpg]

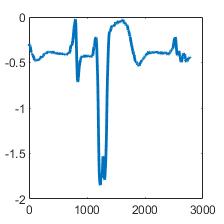

Supplement: S1 Data — (ZIP) [file pone.0253200.s014.zip › Data PlosOne/Ischemic/35038_BL_vessel1_arm_1_cfi60_ECGavg.mat.jpg]

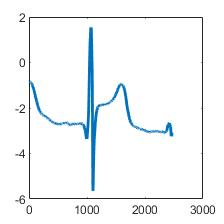

Supplement: S1 Data — (ZIP) [file pone.0253200.s014.zip › Data PlosOne/Ischemic/35038_BL_vessel2_arm_1_cfi60_ECGavg.mat.jpg]

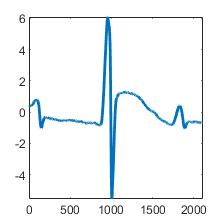

Supplement: S1 Data — (ZIP) [file pone.0253200.s014.zip › Data PlosOne/Ischemic/35038_FU_vessel1_arm_1_cfi60_ECGavg.mat.jpg]

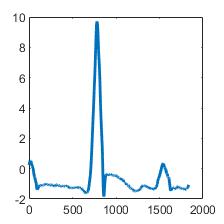

Supplement: S1 Data — (ZIP) [file pone.0253200.s014.zip › Data PlosOne/Ischemic/35038_FU_vessel2_arm_1_cfi60_ECGavg.mat.jpg]

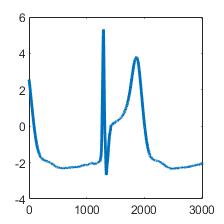

Supplement: S1 Data — (ZIP) [file pone.0253200.s014.zip › Data PlosOne/Ischemic/35039_BL_vessel1_arm_1_cfi60_ECGavg.mat.jpg]

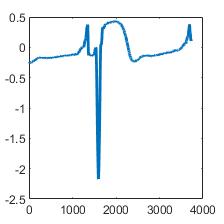

Supplement: S1 Data — (ZIP) [file pone.0253200.s014.zip › Data PlosOne/Ischemic/35039_BL_vessel2_arm_1_cfi60_ECGavg.mat.jpg]

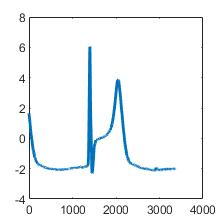

Supplement: S1 Data — (ZIP) [file pone.0253200.s014.zip › Data PlosOne/Ischemic/35039_FU_vessel1_arm_1_cfi60_ECGavg.mat.jpg]

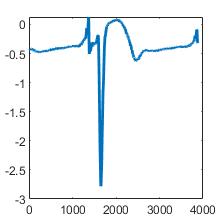

Supplement: S1 Data — (ZIP) [file pone.0253200.s014.zip › Data PlosOne/Ischemic/35039_FU_vessel2_arm_1_cfi60_ECGavg.mat.jpg]

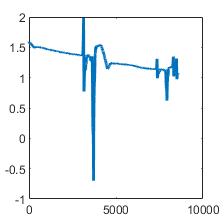

Supplement: S1 Data — (ZIP) [file pone.0253200.s014.zip › Data PlosOne/Ischemic/35040_BL_vessel1_arm_1_cfi60_ECGavg.mat.jpg]

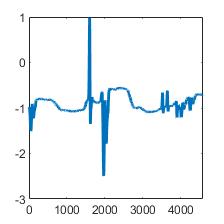

Supplement: S1 Data — (ZIP) [file pone.0253200.s014.zip › Data PlosOne/Ischemic/35040_FU_vessel1_arm_1_cfi60_ECGavg.mat.jpg]

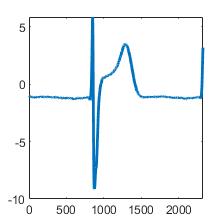

Supplement: S1 Data — (ZIP) [file pone.0253200.s014.zip › Data PlosOne/Ischemic/35041_BL_vessel1_arm_1_cfi60_ECGavg.mat.jpg]

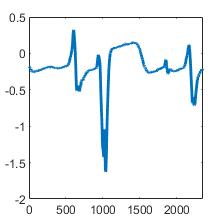

Supplement: S1 Data — (ZIP) [file pone.0253200.s014.zip › Data PlosOne/Ischemic/35041_BL_vessel2_arm_1_cfi60_ECGavg.mat.jpg]

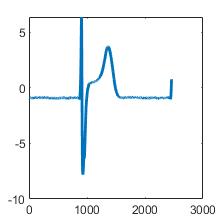

Supplement: S1 Data — (ZIP) [file pone.0253200.s014.zip › Data PlosOne/Ischemic/35041_FU_vessel1_arm_1_cfi60_ECGavg.mat.jpg]

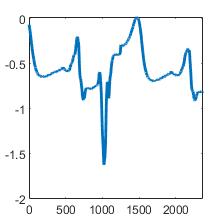

Supplement: S1 Data — (ZIP) [file pone.0253200.s014.zip › Data PlosOne/Ischemic/35041_FU_vessel2_arm_1_cfi60_ECGavg.mat.jpg]

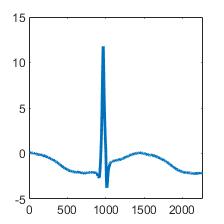

Supplement: S1 Data — (ZIP) [file pone.0253200.s014.zip › Data PlosOne/Ischemic/35042_BL_vessel1_arm_1_cfi60_ECGavg.mat.jpg]

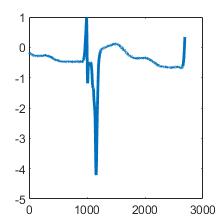

Supplement: S1 Data — (ZIP) [file pone.0253200.s014.zip › Data PlosOne/Ischemic/35042_BL_vessel2_arm_1_cfi60_ECGavg.mat.jpg]

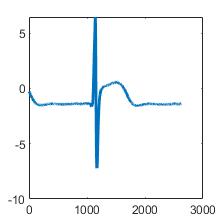

Supplement: S1 Data — (ZIP) [file pone.0253200.s014.zip › Data PlosOne/Ischemic/35042_FU_vessel1_arm_1_cfi60_ECGavg.mat.jpg]

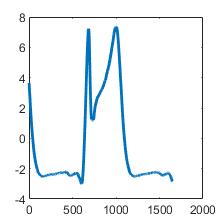

Supplement: S1 Data — (ZIP) [file pone.0253200.s014.zip › Data PlosOne/Ischemic/35043_BL_vessel1_arm_1_cfi60_ECGavg.mat.jpg]

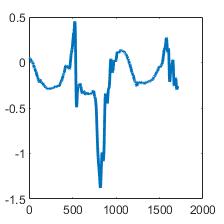

Supplement: S1 Data — (ZIP) [file pone.0253200.s014.zip › Data PlosOne/Ischemic/35043_BL_vessel2_arm_1_cfi60_ECGavg.mat.jpg]

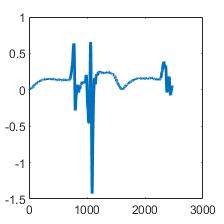

Supplement: S1 Data — (ZIP) [file pone.0253200.s014.zip › Data PlosOne/Ischemic/35044_BL_vessel1_arm_1_cfi60_ECGavg.mat.jpg]

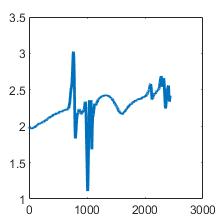

Supplement: S1 Data — (ZIP) [file pone.0253200.s014.zip › Data PlosOne/Ischemic/35044_FU_vessel1_arm_1_cfi60_ECGavg.mat.jpg]

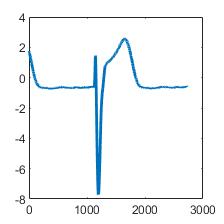

Supplement: S1 Data — (ZIP) [file pone.0253200.s014.zip › Data PlosOne/Ischemic/35046_BL_vessel1_arm_1_cfi60_ECGavg.mat.jpg]

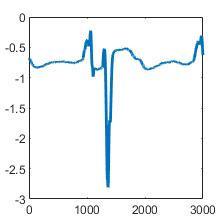

Supplement: S1 Data — (ZIP) [file pone.0253200.s014.zip › Data PlosOne/Ischemic/35046_BL_vessel2_arm_1_cfi60_ECGavg.mat.jpg]

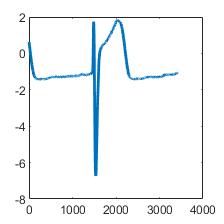

Supplement: S1 Data — (ZIP) [file pone.0253200.s014.zip › Data PlosOne/Ischemic/35046_FU_vessel1_arm_1_cfi60_ECGavg.mat.jpg]

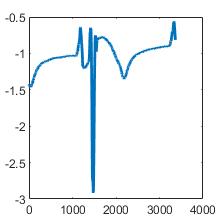

Supplement: S1 Data — (ZIP) [file pone.0253200.s014.zip › Data PlosOne/Ischemic/35046_FU_vessel2_arm_1_cfi60_ECGavg.mat.jpg]

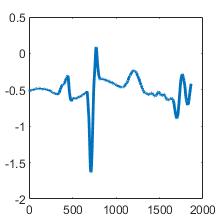

Supplement: S1 Data — (ZIP) [file pone.0253200.s014.zip › Data PlosOne/Ischemic/35047_BL_vessel1_arm_1_cfi60_ECGavg.mat.jpg]

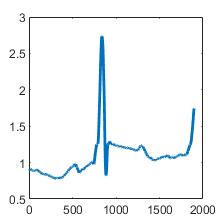

Supplement: S1 Data — (ZIP) [file pone.0253200.s014.zip › Data PlosOne/Ischemic/35047_FU_vessel1_arm_1_cfi60_ECGavg.mat.jpg]

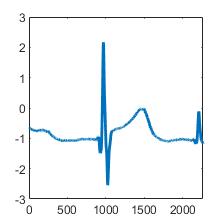

Supplement: S1 Data — (ZIP) [file pone.0253200.s014.zip › Data PlosOne/Ischemic/35048_FU_vessel2_arm_1_cfi60_ECGavg.mat.jpg]

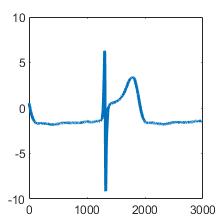

Supplement: S1 Data — (ZIP) [file pone.0253200.s014.zip › Data PlosOne/Ischemic/35049_BL_vessel1_arm_1_cfi60_ECGavg.mat.jpg]

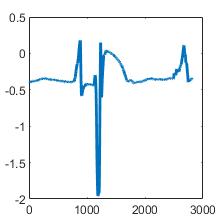

Supplement: S1 Data — (ZIP) [file pone.0253200.s014.zip › Data PlosOne/Ischemic/35049_BL_vessel2_arm_1_cfi60_ECGavg.mat.jpg]

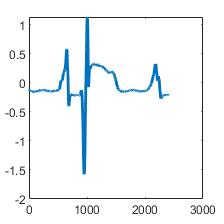

Supplement: S1 Data — (ZIP) [file pone.0253200.s014.zip › Data PlosOne/Ischemic/35049_FU_vessel2_arm_1_cfi60_ECGavg.mat.jpg]

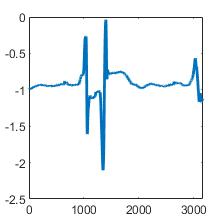

Supplement: S1 Data — (ZIP) [file pone.0253200.s014.zip › Data PlosOne/Ischemic/35050_FU_vessel1_arm_1_cfi60_ECGavg.mat.jpg]

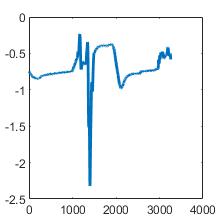

Supplement: S1 Data — (ZIP) [file pone.0253200.s014.zip › Data PlosOne/Ischemic/35051_BL_vessel1_arm_1_cfi60_ECGavg.mat.jpg]

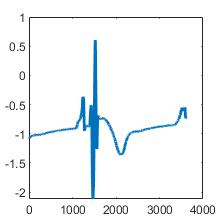

Supplement: S1 Data — (ZIP) [file pone.0253200.s014.zip › Data PlosOne/Ischemic/35051_FU_vessel1_arm_1_cfi60_ECGavg.mat.jpg]

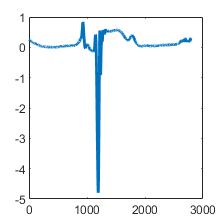

Supplement: S1 Data — (ZIP) [file pone.0253200.s014.zip › Data PlosOne/Ischemic/35052_BL_vessel1_arm_1_cfi60_ECGavg.mat.jpg]

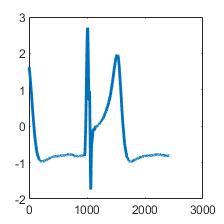

Supplement: S1 Data — (ZIP) [file pone.0253200.s014.zip › Data PlosOne/Ischemic/35052_BL_vessel2_arm_1_cfi60_ECGavg.mat.jpg]

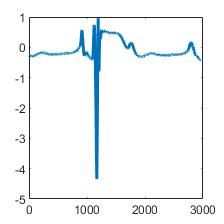

Supplement: S1 Data — (ZIP) [file pone.0253200.s014.zip › Data PlosOne/Ischemic/35052_FU_vessel1_arm_1_cfi60_ECGavg.mat.jpg]

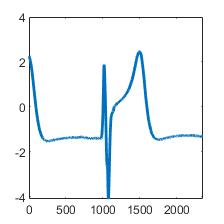

Supplement: S1 Data — (ZIP) [file pone.0253200.s014.zip › Data PlosOne/Ischemic/35052_FU_vessel2_arm_1_cfi60_ECGavg.mat.jpg]

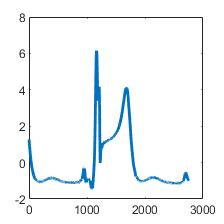

Supplement: S1 Data — (ZIP) [file pone.0253200.s014.zip › Data PlosOne/Ischemic/35053_BL_vessel1_arm_1_cfi60_ECGavg.mat.jpg]

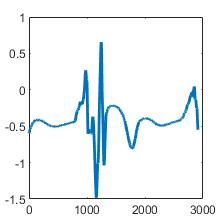

Supplement: S1 Data — (ZIP) [file pone.0253200.s014.zip › Data PlosOne/Ischemic/35053_BL_vessel2_arm_1_cfi60_ECGavg.mat.jpg]

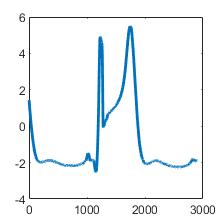

Supplement: S1 Data — (ZIP) [file pone.0253200.s014.zip › Data PlosOne/Ischemic/35053_FU_vessel1_arm_1_cfi60_ECGavg.mat.jpg]

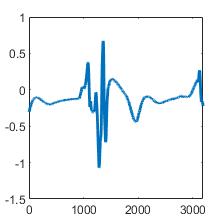

Supplement: S1 Data — (ZIP) [file pone.0253200.s014.zip › Data PlosOne/Ischemic/35053_FU_vessel2_arm_1_cfi60_ECGavg.mat.jpg]

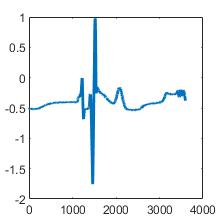

Supplement: S1 Data — (ZIP) [file pone.0253200.s014.zip › Data PlosOne/Ischemic/35054_BL_vessel1_arm_1_cfi60_ECGavg.mat.jpg]

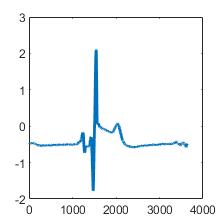

Supplement: S1 Data — (ZIP) [file pone.0253200.s014.zip › Data PlosOne/Ischemic/35054_FU_vessel1_arm_1_cfi60_ECGavg.mat.jpg]

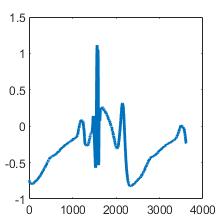

Supplement: S1 Data — (ZIP) [file pone.0253200.s014.zip › Data PlosOne/Ischemic/35055_BL_vessel1_arm_1_cfi60_ECGavg.mat.jpg]

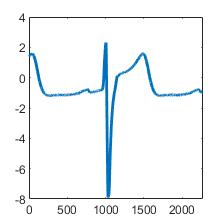

Supplement: S1 Data — (ZIP) [file pone.0253200.s014.zip › Data PlosOne/Ischemic/35057_BL_vessel1_arm_1_cfi60_ECGavg.mat.jpg]

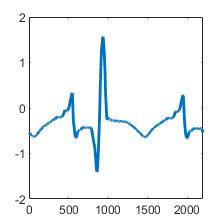

Supplement: S1 Data — (ZIP) [file pone.0253200.s014.zip › Data PlosOne/Ischemic/35059_FU_vessel1_arm_1_cfi60_ECGavg.mat.jpg]

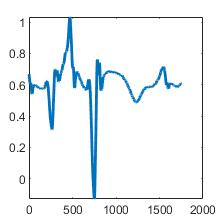

Supplement: S1 Data — (ZIP) [file pone.0253200.s014.zip › Data PlosOne/Ischemic/35060_BL_vessel1_arm_1_cfi60_ECGavg.mat.jpg]

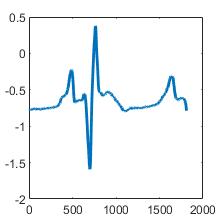

Supplement: S1 Data — (ZIP) [file pone.0253200.s014.zip › Data PlosOne/Ischemic/35060_FU_vessel1_arm_1_cfi60_ECGavg.mat.jpg]

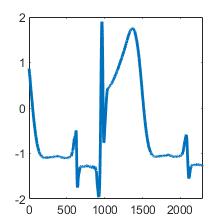

Supplement: S1 Data — (ZIP) [file pone.0253200.s014.zip › Data PlosOne/Ischemic/35061_BL_vessel1_arm_1_cfi60_ECGavg.mat.jpg]

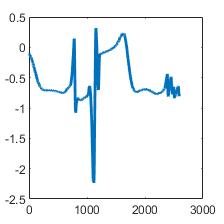

Supplement: S1 Data — (ZIP) [file pone.0253200.s014.zip › Data PlosOne/Ischemic/35061_FU_vessel1_arm_1_cfi60_ECGavg.mat.jpg]

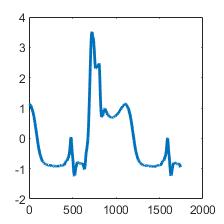

Supplement: S1 Data — (ZIP) [file pone.0253200.s014.zip › Data PlosOne/Ischemic/35062_BL_vessel1_arm_1_cfi60_ECGavg.mat.jpg]

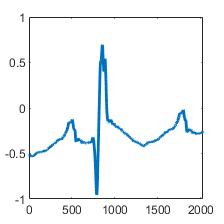

Supplement: S1 Data — (ZIP) [file pone.0253200.s014.zip › Data PlosOne/Ischemic/35062_BL_vessel2_arm_1_cfi60_ECGavg.mat.jpg]

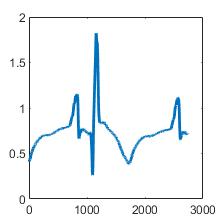

Supplement: S1 Data — (ZIP) [file pone.0253200.s014.zip › Data PlosOne/Ischemic/35062_FU_vessel2_arm_1_cfi60_ECGavg.mat.jpg]

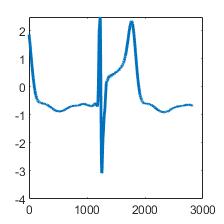

Supplement: S1 Data — (ZIP) [file pone.0253200.s014.zip › Data PlosOne/Ischemic/35062_FU_vessel3_arm_1_cfi60_ECGavg.mat.jpg]

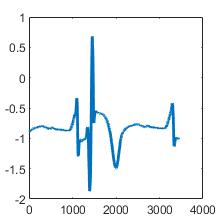

Supplement: S1 Data — (ZIP) [file pone.0253200.s014.zip › Data PlosOne/Ischemic/35063_BL_vessel1_arm_1_cfi60_ECGavg.mat.jpg]

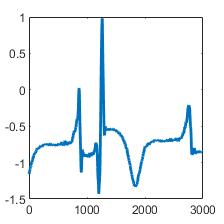

Supplement: S1 Data — (ZIP) [file pone.0253200.s014.zip › Data PlosOne/Ischemic/35063_FU_vessel1_arm_1_cfi60_ECGavg.mat.jpg]

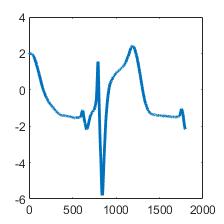

Supplement: S1 Data — (ZIP) [file pone.0253200.s014.zip › Data PlosOne/Ischemic/35064_BL_vessel1_arm_1_cfi60_ECGavg.mat.jpg]

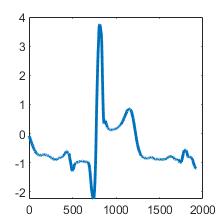

Supplement: S1 Data — (ZIP) [file pone.0253200.s014.zip › Data PlosOne/Ischemic/35064_BL_vessel2_arm_1_cfi60_ECGavg.mat.jpg]

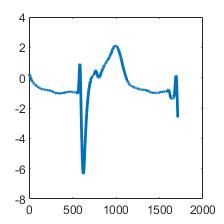

Supplement: S1 Data — (ZIP) [file pone.0253200.s014.zip › Data PlosOne/Ischemic/35064_FU_vessel1_arm_1_cfi60_ECGavg.mat.jpg]

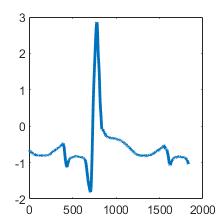

Supplement: S1 Data — (ZIP) [file pone.0253200.s014.zip › Data PlosOne/Ischemic/35064_FU_vessel2_arm_1_cfi60_ECGavg.mat.jpg]

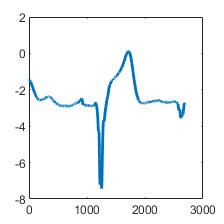

Supplement: S1 Data — (ZIP) [file pone.0253200.s014.zip › Data PlosOne/Ischemic/35065_BL_vessel1_arm_1_cfi60_ECGavg.mat.jpg]

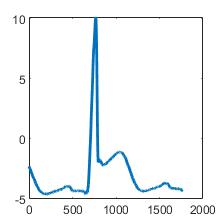

Supplement: S1 Data — (ZIP) [file pone.0253200.s014.zip › Data PlosOne/Ischemic/35065_FU_vessel2_arm_1_cfi60_ECGavg.mat.jpg]

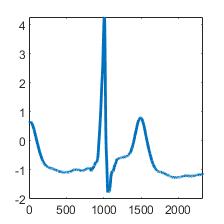

Supplement: S1 Data — (ZIP) [file pone.0253200.s014.zip › Data PlosOne/Ischemic/35066_BL_vessel1_arm_1_cfi60_ECGavg.mat.jpg]

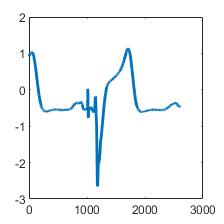

Supplement: S1 Data — (ZIP) [file pone.0253200.s014.zip › Data PlosOne/Ischemic/35066_BL_vessel2_arm_1_cfi60_ECGavg.mat.jpg]

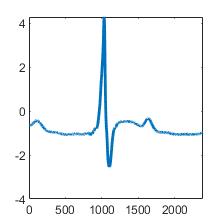

Supplement: S1 Data — (ZIP) [file pone.0253200.s014.zip › Data PlosOne/Ischemic/35066_FU_vessel1_arm_1_cfi60_ECGavg.mat.jpg]

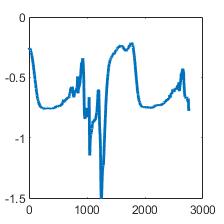

Supplement: S1 Data — (ZIP) [file pone.0253200.s014.zip › Data PlosOne/Ischemic/35066_FU_vessel2_arm_1_cfi60_ECGavg.mat.jpg]

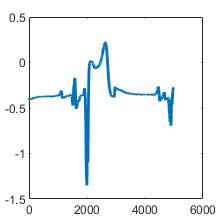

Supplement: S1 Data — (ZIP) [file pone.0253200.s014.zip › Data PlosOne/Ischemic/35067_BL_vessel1_arm_1_cfi60_ECGavg.mat.jpg]

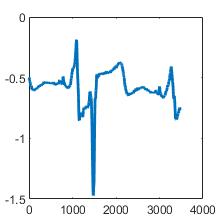

Supplement: S1 Data — (ZIP) [file pone.0253200.s014.zip › Data PlosOne/Ischemic/35067_FU_vessel1_arm_1_cfi60_ECGavg.mat.jpg]

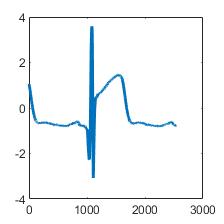

Supplement: S1 Data — (ZIP) [file pone.0253200.s014.zip › Data PlosOne/Ischemic/35068_BL_vessel1_arm_1_cfi60_ECGavg.mat.jpg]

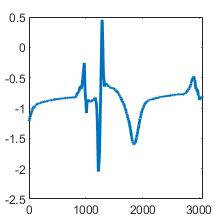

Supplement: S1 Data — (ZIP) [file pone.0253200.s014.zip › Data PlosOne/Ischemic/35069_FU_vessel1_arm_1_cfi60_ECGavg.mat.jpg]

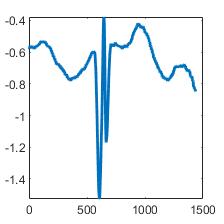

Supplement: S1 Data — (ZIP) [file pone.0253200.s014.zip › Data PlosOne/Ischemic/35070_BL_vessel1_arm_1_cfi60_ECGavg.mat.jpg]

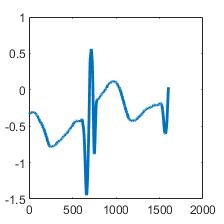

Supplement: S1 Data — (ZIP) [file pone.0253200.s014.zip › Data PlosOne/Ischemic/35070_FU_vessel1_arm_1_cfi60_ECGavg.mat.jpg]

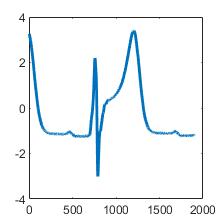

Supplement: S1 Data — (ZIP) [file pone.0253200.s014.zip › Data PlosOne/Ischemic/35071_BL_vessel1_arm_1_cfi60_ECGavg.mat.jpg]

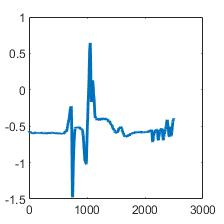

Supplement: S1 Data — (ZIP) [file pone.0253200.s014.zip › Data PlosOne/Ischemic/35071_FU_vessel1_arm_1_cfi60_ECGavg.mat.jpg]

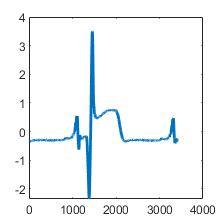

Supplement: S1 Data — (ZIP) [file pone.0253200.s014.zip › Data PlosOne/Ischemic/35072_BL_vessel1_arm_1_cfi60_ECGavg.mat.jpg]

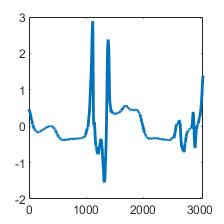

Supplement: S1 Data — (ZIP) [file pone.0253200.s014.zip › Data PlosOne/Ischemic/35072_FU_vessel1_arm_1_cfi60_ECGavg.mat.jpg]

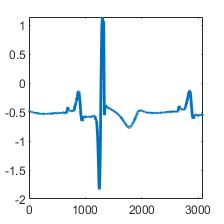

Supplement: S1 Data — (ZIP) [file pone.0253200.s014.zip › Data PlosOne/Ischemic/35073_BL_vessel1_arm_1_cfi60_ECGavg.mat.jpg]

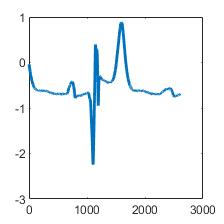

Supplement: S1 Data — (ZIP) [file pone.0253200.s014.zip › Data PlosOne/Ischemic/35073_FU_vessel1_arm_1_cfi60_ECGavg.mat.jpg]

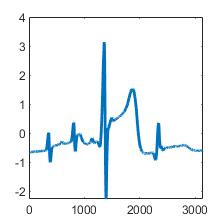

Supplement: S1 Data — (ZIP) [file pone.0253200.s014.zip › Data PlosOne/Ischemic/35073_FU_vessel2_arm_1_cfi60_ECGavg.mat.jpg]

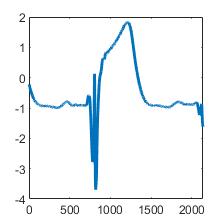

Supplement: S1 Data — (ZIP) [file pone.0253200.s014.zip › Data PlosOne/Ischemic/35074_BL_vessel1_arm_1_cfi60_ECGavg.mat.jpg]

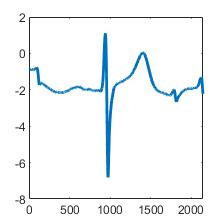

Supplement: S1 Data — (ZIP) [file pone.0253200.s014.zip › Data PlosOne/Ischemic/35074_BL_vessel2_arm_1_cfi60_ECGavg.mat.jpg]

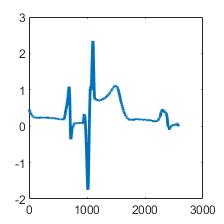

Supplement: S1 Data — (ZIP) [file pone.0253200.s014.zip › Data PlosOne/Ischemic/35074_FU_vessel1_arm_1_cfi60_ECGavg.mat.jpg]

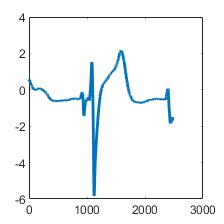

Supplement: S1 Data — (ZIP) [file pone.0253200.s014.zip › Data PlosOne/Ischemic/35074_FU_vessel2_arm_1_cfi60_ECGavg.mat.jpg]

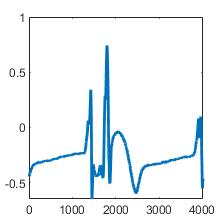

Supplement: S1 Data — (ZIP) [file pone.0253200.s014.zip › Data PlosOne/Ischemic/35075_BL_vessel1_arm_1_cfi60_ECGavg.mat.jpg]

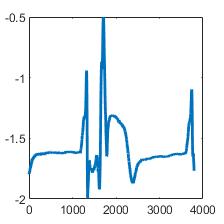

Supplement: S1 Data — (ZIP) [file pone.0253200.s014.zip › Data PlosOne/Ischemic/35075_FU_vessel1_arm_1_cfi60_ECGavg.mat.jpg]

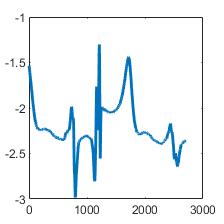

Supplement: S1 Data — (ZIP) [file pone.0253200.s014.zip › Data PlosOne/Ischemic/35077_FU_vessel1_arm_1_cfi60_ECGavg.mat.jpg]

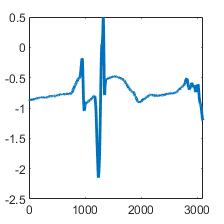

Supplement: S1 Data — (ZIP) [file pone.0253200.s014.zip › Data PlosOne/Ischemic/35078_FU_vessel1_arm_1_cfi60_ECGavg.mat.jpg]

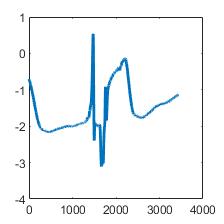

Supplement: S1 Data — (ZIP) [file pone.0253200.s014.zip › Data PlosOne/Ischemic/35079_BL_vessel1_arm_1_cfi60_ECGavg.mat.jpg]

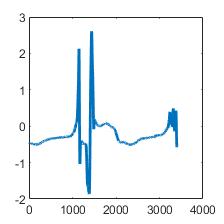

Supplement: S1 Data — (ZIP) [file pone.0253200.s014.zip › Data PlosOne/Ischemic/35079_FU_vessel1_arm_1_cfi60_ECGavg.mat.jpg]

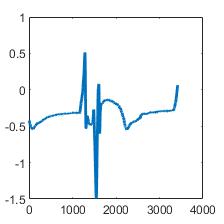

Supplement: S1 Data — (ZIP) [file pone.0253200.s014.zip › Data PlosOne/Ischemic/35080_BL_vessel1_arm_1_cfi60_ECGavg.mat.jpg]

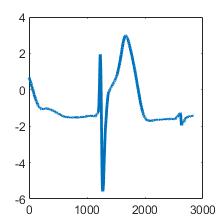

Supplement: S1 Data — (ZIP) [file pone.0253200.s014.zip › Data PlosOne/Ischemic/35080_BL_vessel2_arm_1_cfi60_ECGavg.mat.jpg]

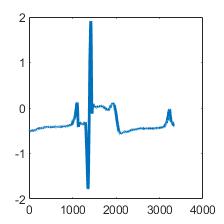

Supplement: S1 Data — (ZIP) [file pone.0253200.s014.zip › Data PlosOne/Ischemic/35080_FU_vessel1_arm_1_cfi60_ECGavg.mat.jpg]

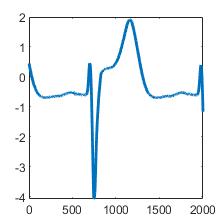

Supplement: S1 Data — (ZIP) [file pone.0253200.s014.zip › Data PlosOne/Ischemic/35080_FU_vessel2_arm_1_cfi60_ECGavg.mat.jpg]

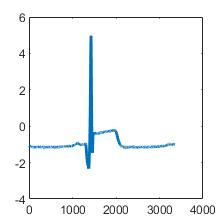

Supplement: S1 Data — (ZIP) [file pone.0253200.s014.zip › Data PlosOne/Ischemic/35080_FU2_vessel1_arm_1_cfi60_ECGavg.mat.jpg]
